# Systematic evaluation of chromosome conformation capture assays

**DOI:** 10.1038/s41592-021-01248-7

**Published:** 2021-09-03

**Authors:** Betul Akgol Oksuz, Liyan Yang, Sameer Abraham, Sergey V. Venev, Nils Krietenstein, Krishna Mohan Parsi, Hakan Ozadam, Marlies E. Oomen, Ankita Nand, Hui Mao, Ryan M. J. Genga, Rene Maehr, Oliver J. Rando, Leonid A. Mirny, Johan H. Gibcus, Job Dekker

**Affiliations:** 1 Program in Systems Biology, Department of Biochemistry and Molecular Pharmacology, University of Massachusetts Medical School, Worcester, MA USA; 2 Department of Physics, Massachusetts Institute of Technology, Cambridge, MA USA; 3 Department of Biochemistry and Molecular Pharmacology, University of Massachusetts Medical School, Worcester, MA USA; 4 Program in Molecular Medicine, University of Massachusetts Medical School, Worcester, MA USA; 5 Program in Molecular Medicine, Diabetes Center of Excellence, University of Massachusetts Medical School, Worcester, MA USA; 6 Department of Molecular Biosciences, University of Texas at Austin, Austin, TX USA; 7 Institute for Medical Engineering and Science, Massachusetts Institute of Technology, Cambridge, MA USA; 8 Graduate Program in Biophysics, Harvard University, Cambridge, MA USA; 9 Howard Hughes Medical Institute, Chevy Chase, MD USA

**Keywords:** Genomic analysis, Genomics

## Abstract

Chromosome conformation capture (3C) assays are used to map chromatin interactions genome-wide. Chromatin interaction maps provide insights into the spatial organization of chromosomes and the mechanisms by which they fold. Hi-C and Micro-C are widely used 3C protocols that differ in key experimental parameters including cross-linking chemistry and chromatin fragmentation strategy. To understand how the choice of experimental protocol determines the ability to detect and quantify aspects of chromosome folding we have performed a systematic evaluation of 3C experimental parameters. We identified optimal protocol variants for either loop or compartment detection, optimizing fragment size and cross-linking chemistry. We used this knowledge to develop a greatly improved Hi-C protocol (Hi-C 3.0) that can detect both loops and compartments relatively effectively. In addition to providing benchmarked protocols, this work produced ultra-deep chromatin interaction maps using Micro-C, conventional Hi-C and Hi-C 3.0 for key cell lines used by the 4D Nucleome project.

## Abstract (web summary)

This analysis systematically evaluates cross-linking chemistry and chromatin fragmentation strategies commonly used in 3C assays and introduces an improved Hi-C protocol for detecting loops and compartments.

## Main

Chromosome conformation capture (3C)-based assays^1^ have become widely used to generate genome-wide chromatin interaction maps^2^. Analysis of chromatin interaction maps has led to detection of several features of the folded genome. Such features include precise looping interactions (at the 0.1–1 Mb scale) between pairs of specific sites that appear as local dots in interaction maps. Many of such dots represent loops formed by cohesin-mediated loop extrusion that is stalled at convergent CCCTC-binding factor (CTCF) sites^3–5^. Loop extrusion also produces other features in interaction maps such as stripe-like patterns anchored at specific sites that block loop extrusion. The effective depletion of interactions across such blocking sites leads to domain boundaries (insulation). At the megabase scale, interaction maps of many organisms including mammals display checkerboard patterns that represent the spatial compartmentalization of two main types of chromatin: active and open A-type chromatin domains, and inactive and more closed B-type chromatin domains^6^.

The Hi-C protocol has evolved over the years. While initial protocols used restriction enzymes such as HindIII that produces relatively large fragments of several kilobases^6^, over the last 5 years Hi-C using DpnII or MboI digestion has become the protocol of choice for mapping chromatin interactions at kilobase resolution^3^. More recently, Micro-C, which uses MNase instead of restriction enzymes as well as a different cross-linking protocol, was shown to allow generation of nucleosome-level interaction maps^7–9^. It is critical to ascertain how key parameters of these 3C-based methods, including cross-linking and chromatin fragmentation, quantitatively influence the detection of chromatin interaction frequencies and the detection of different chromosome folding features that range from local looping between small intra-chromosomal (cis) elements to global compartmentalization of megabase-sized domains. Here, we systematically assessed how different cross-linking and fragmentation methods yield quantitatively different chromatin interaction maps.

## Results

**Fig. 1:**
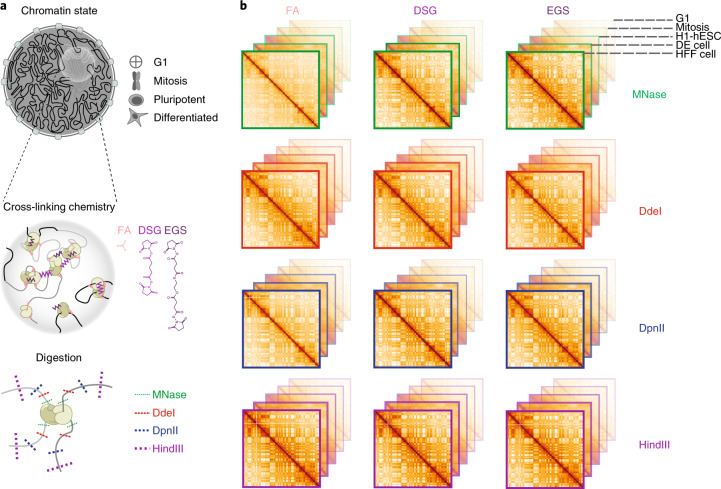
Outline of the experimental design. **a**, Experimental design for conformation capture for various cells, cross-linkers and enzymes. **b**, Representation of interaction maps from experiments in **a**.

**Extended Data Fig. 1:**
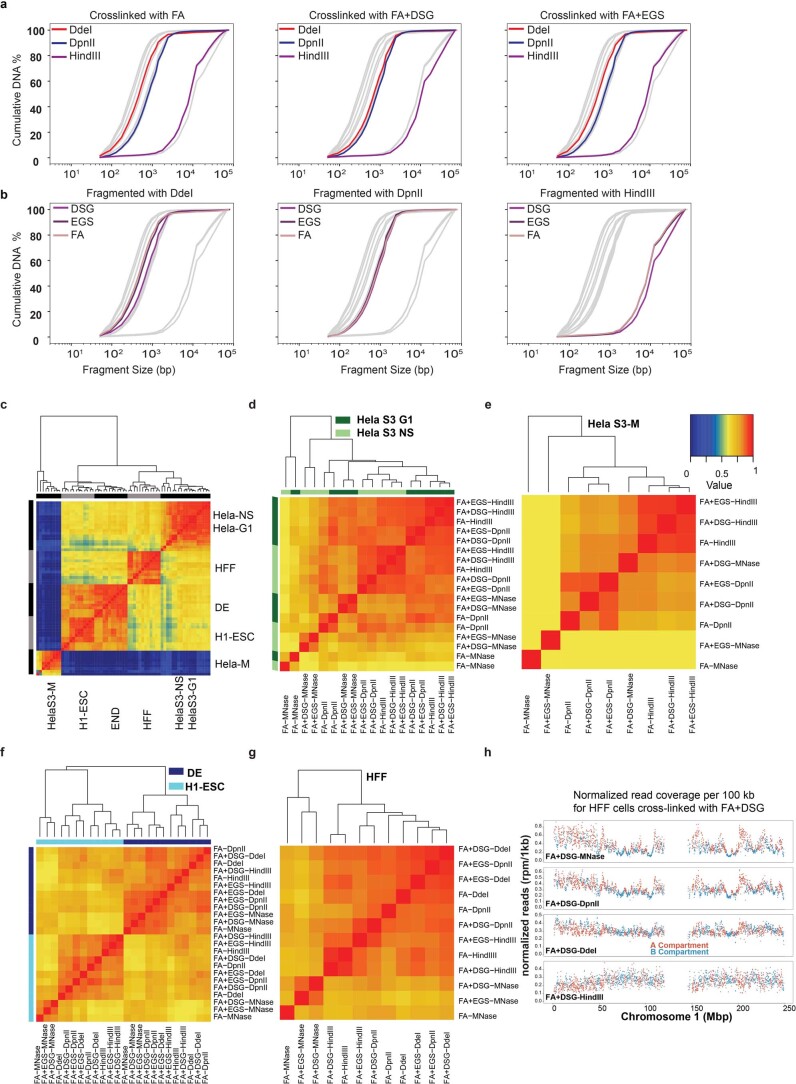
DNA fragmentation and clustering of correlation (HiCRep). **a,b**. Cumulative distribution of the lengths of fragmented DNA obtained from fragment analyzer data in HFF cells stratified for different cross-linkers (a) and restriction enzymes (b). Gray lines indicate all datasets, colored lines indicate data obtained with the indicated nuclease/cross-linkers. **c-g**. Hierarchical clustering of HiCRep correlations for: all protocols comparing cell states (c), synchronized HeLa-S3 G1 cells (dark green) and non-synchronized HeLa-S3 cells (light green) (d), synchronized HeLa-S3 mitotic cells (e), H1-hESC and H1-hESC derived DE cells (f), 12 protocols applied to HFF cells (g). One color key is indicated for all of the heatmaps. **h**. Genome coverage of data generated using MNase, DdeI, DpnII and HindIII. The read density was normalized to reads per million, separated by the coverage in A and B compartments (Methods). Source data

We explored how two key parameters of 3C-based protocols, cross-linking and chromatin fragmentation, determine the ability to quantitatively detect chromatin compartment domains and loops. We selected three cross-linkers widely used for chromatin: 1% formaldehyde (FA), conventional for most 3C-based protocols; 1% FA followed by incubation with 3 mM disuccinimidyl glutarate (the FA + DSG protocol); and 1% FA followed by incubation with 3 mM ethylene glycol bis(succinimidylsuccinate) (the FA + EGS protocol) (Fig. 1a). We selected four different nucleases for chromatin fragmentation: MNase, DdeI, DpnII and HindIII, which fragment chromatin in sizes ranging from single nucleosomes to multiple kilobases. Combined, the three cross-linking and four fragmentation strategies yield a matrix of 12 distinct protocols (Fig. 1b). To determine how performance of these protocols varies for different states of chromatin we applied this matrix of protocols to multiple cell types and cell cycle stages. We analyzed four different cell types: pluripotent H1 human embryonic stem cells (H1-hESCs), differentiated endoderm (DE) cells derived from H1-hESCs, fully differentiated human foreskin fibroblast (HFF) cells (12 protocols for each), and HeLa-S3 cells (9 protocols). We analyzed two cell cycle stages: G1 and mitosis, in HeLa-S3 cells (9 protocols for each; Fig. 1). Each interaction library was then sequenced on a single lane of a HiSeq4000 instrument, producing ~150–200 million uniquely mapping read pairs (Supplementary Table 1). We used the Distiller pipeline to align the sequencing reads, and pairtools and cooler^10^ packages to process mapped reads and create multi-resolution contact maps (Methods). Given that the density of restriction sites for DdeI, DpnII and HindIII fluctuates along chromosomes, we observed different read coverages in raw interaction maps obtained from datasets using these enzymes (Extended Data Fig. 1h). These differences were removed after matrix balancing^11^.

We first assessed the size range of the chromatin fragments produced after digestion by the 12 protocols for HFF cells (Methods). Digestion with HindIII resulted in 5–20-kb DNA fragments; DpnII and DdeI produced fragments of 0.5–5 kb; and MNase protocols included a size selection step to ensure that the ligation product involved two mononucleosome-sized fragments (~150 bp) (Extended Data Fig. 1). Different cross-linkers did not affect the size ranges produced by the different nucleases, although DSG cross-linking lowered digestion efficiency slightly (Extended Data Fig. 1b).

### All 3C-based protocols can differentiate between cell states

We first assessed the similarity between the 63 datasets by global and pairwise correlations using HiCRep and hierarchical clustering (Extended Data Fig. 1c)^12,13^. We found that the datasets are highly correlated and cluster primarily by cell type and state and then by cell type similarity, for example H1-hESCs and H1-hESC-derived DE cells cluster together; and the most distinct cluster is formed by mitotic HeLa cells. MNase protocols show slightly lower correlations with Hi-C experiments.

### Extra cross-linking yields more intra-chromosomal contacts

**Fig. 2:**
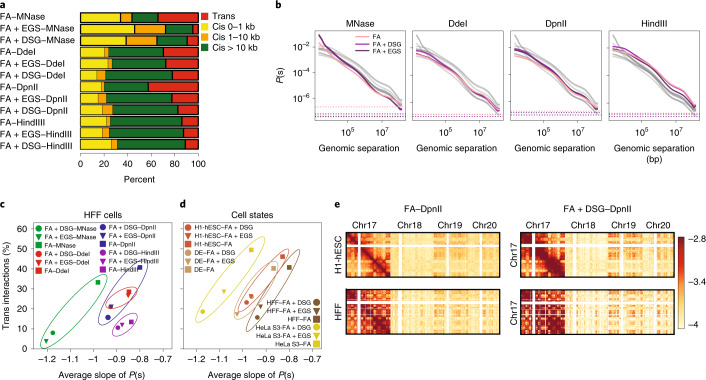
Extra cross-linking yields more intra-chromosomal contacts. **a**, The number of valid pairs in each of the 12 HFF protocols by genomic distance. **b**, Distance-dependent contact probability for the 12 HFF protocols. Each plot shows *P*(s) for experiments performed with the indicated nuclease. The colored lines indicate the cross-linkers and the gray lines indicate all datasets. The dashed lines show the level of the trans interactions. **c**, The percentage of trans interactions versus the average slope of distance-dependent contact probability for all cross-linker and enzyme combinations in HFF. Nucleases are grouped by colored ovals. **d**, Percentage of trans interactions versus the average slope of distance-dependent contact probability for each cell state. Only experiments cross-linked with FA, FA + DSG or FA + EGS and digested with DpnII are shown. **e**, Interactions (log transformed) between chromosome (chr) 17 and chromosomes 17, 18, 19 and 20 for FA or FA + DSG cross-linking and DpnII digestion, in H1-hESC and HFF cells. Total trans interactions for FA–DpnII protocols in H1-hESCs, 47.7%; HFF cells, 42.5%; and for FA + DSG–DpnII protocols in H1-hESCs, 25%; and HFF cells, 17.3%. Source data

**Extended Data Fig. 2:**
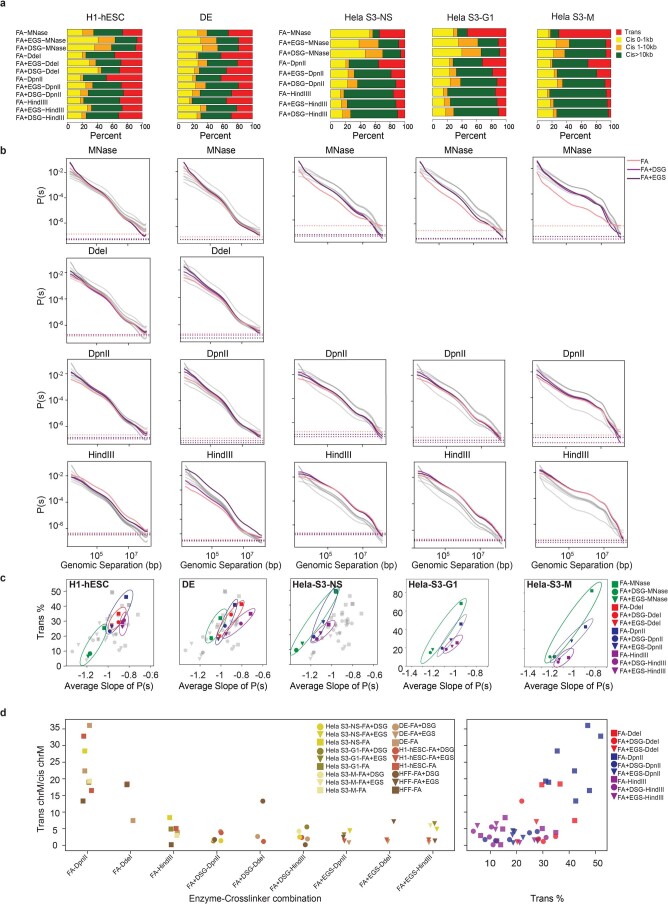
Cis and trans contact frequency differ between protocols. **a**. The number of valid pairs in each of the 12 protocols applied to H1-hESC, DE, HeLa-S3-NS, HeLa-S3-G1 and HeLa-S3-M cells partitioned by genomic distances. **b**. Distance dependent contact probability of 12 protocols ordered as in (a), partitioned by fragmenting nucleases used (gray lines indicate all datasets, colored lines indicate datasets generated with the nucleases indicated for each plot). **c**. The relationship between the trans percent and the average slope of the distance dependent contact probability for the 12 protocols ordered as in Extended Data Fig. 2a. d. Quantification of protocol introduced noise as defined by inter-mitochondrial interactions (chrM with chr1-22), normalized by intra-mitochondrial (chrM with chrM) interactions. Source data

Given that chromosomes occupy individual territories, intra-chromosomal (cis) interactions are more frequent than inter-chromosomal (trans) interactions^14^. The cis : trans ratio is commonly used as an indicator of Hi-C library quality given that inter-chromosomal interactions are a mixture of true chromatin interactions and interactions that are the result of random ligations^14,15^. For all enzymes and cell types, we found that the addition of DSG or EGS to FA cross-linking decreased the percentage of trans interactions (Fig. 2a for HFF and Extended Data Fig. 2a for H1-hESC, DE, HeLa-S3).

Regarding intra-chromosomal interactions, we noticed two distinct patterns. First, digestion into smaller fragments increased short-range interactions. MNase digestion generated more interactions between loci separated by less than 10 kb, whereas digestion with either DdeI, DpnII or HindIII resulted in a relatively larger number of interactions between loci separated by more than 10 kb (Fig. 2a,b for HFF and Extended Data Fig. 2a,b for DE, H1-hESC, HeLa-S3). Second, *P*(*s*) plots showed that the addition of either DSG or EGS resulted in a steeper decay in interaction frequency as a function of genomic distance for all fragmentation protocols. Moreover, for a given chromatin fragmentation level, additional cross-linking with DSG or EGS reduced trans interactions, as shown for HFF cells and all other cell types and cell stages studied (Fig. 2c,d and Extended Data Fig. 2c). The addition of DSG or EGS could have reduced fragment mobility and the formation of spurious ligations, resulting in a steeper slope of the *P*(*s*). We note a difference in slopes for data obtained with different cell types and cell cycle stages, which could reflect state-dependent differences in chromatin compaction.

Random ligation events between un-cross-linked, freely diffusing fragments lead to noise that is mostly seen in trans and long-range cis interactions. Experiments that use DpnII and additional cross-linkers have a general decrease in trans contacts, while uncovering a stronger trans compartmental pattern (Fig. 2e). Additionally, the comparison of trans interaction frequencies to interactions between mitochondrial and nuclear genomes, given that these interactions can result only from random ligations (Extended Data Fig. 2d), showed that random ligations between genomic and mitochondrial DNA were the lowest when chromatin was fragmented with HindIII, and were generally higher when chromatin was fragmented into smaller segments. Additional DSG or EGS cross-linking reduced random ligation in experiments using DpnII or DdeI. We could not use this noise metric for experiments using MNase because MNase completely degrades the mitochondrial genome.

### Fragment size and cross-linking affect compartment strength

**Fig. 3:**
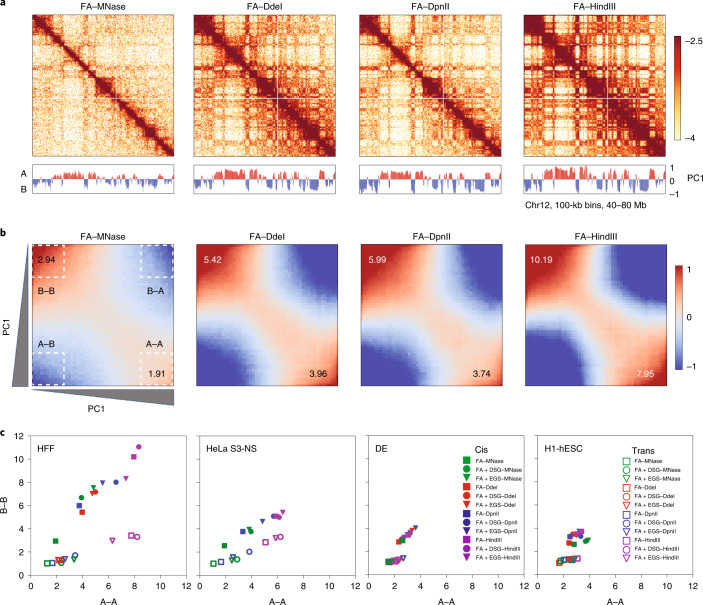
Fragment size and cross-linking affect compartment strength. **a**, Interactions (log transformed) for HFF cells obtained after cross-linking with FA only and digestion with MNase, DdeI, DpnII and HindIII, respectively. Principal component 1 (PC1) values of the genomic region are displayed below the figure. **b**, Saddle plots of genome-wide interaction maps for data shown in **a**. The signal of A–A and B–B compartmentalization in cis interactions become stronger with increasing fragment size. **c**, Quantification of the compartment strength using saddle plots of cis and trans interactions for 12 protocols applied to HFF cells, 9 protocols to non-synchronized HeLa-S3 (HeLa-S3 NS) NS, 12 protocols to DE cells, and 12 protocols to H1-hESCs. The *y* axis represents the strongest 20% of B–B interactions and the *x* axis represents the strongest 20% of A–A interactions, normalized by the bottom 20% of A–B interactions; that is, *y* = top(B–B)/bottom(A–B) and *x* = top(A–A)/bottom(A–B). Source data

Visual inspection of interaction matrices (binned at 100-kb resolution) suggested that the contrast between the domains that comprise the A and B compartments can vary between protocols. For instance, for HFF cells cross-linked with only FA, interaction matrices obtained with MNase digestion displayed a relatively weak compartment pattern, whereas those obtained with HindIII digestion had much stronger patterns (Fig. 3a).

**Extended Data Fig. 3:**
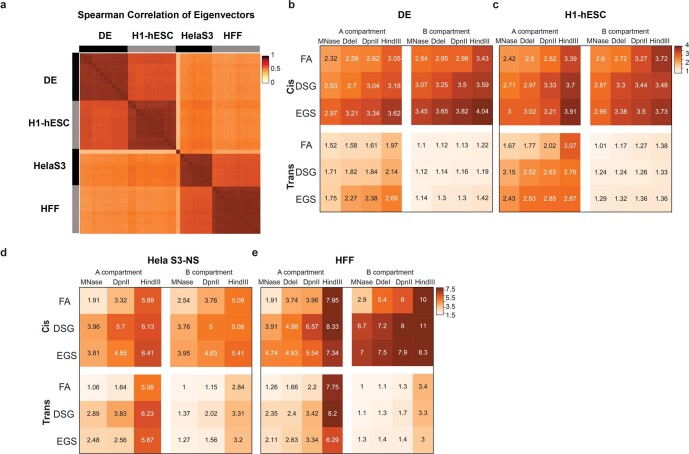
Quantitative compartment detection differ between protocols. **a**. Hierarchical clustering of Spearman correlations of Eigenvectors (PC1) for 63 protocols. Clustering shows strong correlations between compartments from data obtained with varying protocols applied to the same cell types and weaker correlations for data obtained with the same protocols applied to different cell types. **b–e**. A-A and B-B compartment strength of saddle plots for fixation versus enzyme stratified by cell state: DE (b), H1-hESC (c), HeLa-S3-NS (d), HFF (e). For each cell type, saddle plot quantification was done for cis and trans reads separately. Source data

To investigate compartmentalization and determine the positions of A and B compartments, we used eigenvector decomposition^6,11^ for all cell states except for mitotic cells, given that they do not display compartmentalized chromosomes^16^. Correlation between compartment profiles of all experiments showed that the greatest difference in profiles can be attributed to cell type (Extended Data Fig. 3a). Within each cell type, positions of compartment domains obtained with different protocols were highly similar (Spearman correlation > 0.8; Extended Data Fig. 3a).

Compartment strength analysis using saddle plots (Methods^11,17,18^) revealed three important trends. First, protocols that generate larger fragments (for example using HindIII; Fig. 3b,c) and protocols that include additional DSG or EGS cross-linking produced quantitatively stronger compartment patterns (Fig. 3c and Extended Data Fig. 3b–e) for all four cell types. Second, the different cell types differed in compartment strength: HFF cells displayed the strongest compartment pattern, while H1-hESCs displayed the weakest compartment pattern regardless of the protocol used. This could be related to differences in chromatin state and/or cell cycle distribution between the cell types. Last, compartment strength was much stronger in cis than in trans. Furthermore, some protocols, including the conventional Hi-C protocol (cross-linked with FA and digestion with DpnII) and MNase-based protocols (Micro-C, regardless of cross-linking protocol) did not detect enrichment of B–B interactions between chromosomes (Extended Data Fig. 3). Such preferred B–B interactions were detected only when Hi-C was performed with HindIII (Extended Data Fig. 3d,e). Additionally, trans preferential A–A interactions were more frequent than trans preferential B–B interactions for all protocols and cell types. In summary, compartment strength was stronger both in cis and in trans, when protocols produce larger fragments or use additional cross-linking.

### Fragment size and cross-linking determine loop detection

**Extended Data Fig. 4:**
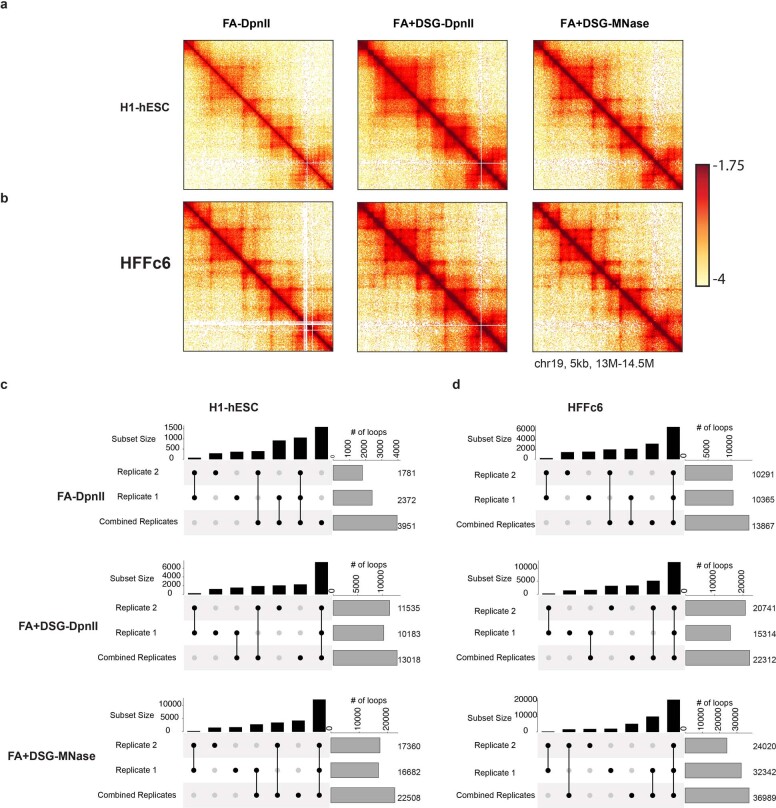
FA+DSG cross-linking produces reproducible chromatin loops. **a**. Interaction heatmaps (log transformed) of experiments for H1-ESC cells obtained from the following cross-linker-enzyme combinations (from left to right): FA-DpnII, FA+DSG-DpnII and FA+DSG-MNase. **b**. Interaction heatmaps of protocols specified in Extended Data Fig. 4a for HFFc6 cells. **c**.Upset plots of loops detected with different replicates for H1-hESC show: 1) total number of loops detected in Replicate 1, Replicate 2 and merged replicates on the right side (gray bars), 2) number of loops detected in the one, two or three experiments shown in black bars. Loops found with only one or multiple experiments are highlighted and connected with black dots. Here Upset plots investigate the consistency of loops between each of the replicates and combined replicates for FA-DpnII, FA+DSG-DpnII and FA+DSG-MNase in H1-hESC. **d**. Upset plots (as explained in Extended Data Fig. 4c) of loops detected with different replicates for HFFc6 cells.

Of all of the structural Hi-C features, the detection of loops depends most on sequencing depth. We applied conventional Hi-C using FA and DpnII digestion (FA–DpnII); Hi-C using DSG in addition to FA cross-linking and DpnII digestion (FA + DSG–DpnII); and the standard Micro-C protocol (FA + DSG–MNase) to two cell types, H1-hESC and HFFc6, and sequenced these libraries to a depth of 2.4–3.9 billion valid interactions. HFFc6 is a subclone of HFF cells and is used by the 4D Nucleome Consortium^19^. Interaction maps of data obtained from these ‘deep’ datasets showed quantitative differences in interactions for both H1-hESC and HFFc6 (Extended Data Fig. 4a,b). As compared with the conventional Hi-C protocol, the use of additional DSG cross-linking and finer fragmentation produced contact maps with more contrast and more pronounced focal enrichment of specific looping contacts. We re-implemented HICCUPS to identify looping interactions that appear as dots^3,8^ (Methods).

**Fig. 4:**
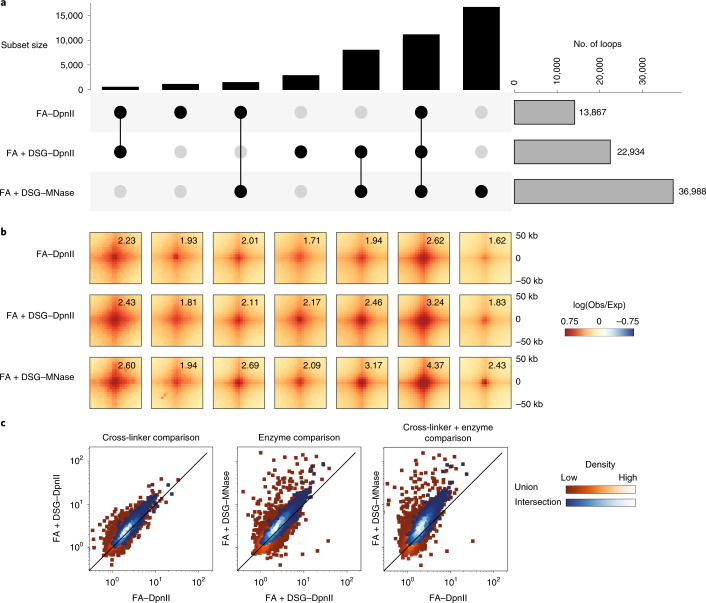
Fragment size and cross-linking determine loop detection. **a**, ‘Upset’ plot of loops detected in protocols using HFFc6 cells showing the total number of loops detected in the FA–DpnII, FA + DSG–DpnII and FA + DSG–MNase protocols on the right side (gray bars), and the number of loops detected in one, two or three of these protocols (shown in black bars). Loops found with one or multiple protocols are connected with black dots. **b**, Pileups of the loops shown in **a**. Numbers in each pileup represent signal enrichment at the loop compared with local background (Methods). **c**, Scatter plots for the strength of individual loops calculated in the same way as in **b** between protocol pairs in HFFc6 cells. The plots display two sets of looping interactions: the union sets (red squares) and the intersection sets (blue circles) from the three protocols. The color scale represents the density of loop interactions. Source data

**Extended Data Fig. 5:**
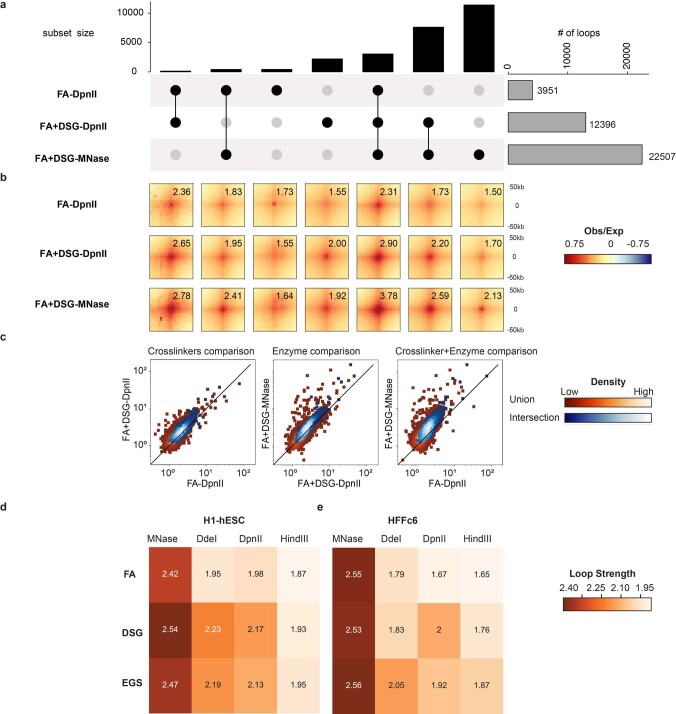
Fine fragmentation and DSG cross-linking improves loop detection. **a**. Loops for HFFc6 shows the 1) total number of loops detected with FA-DpnII, FA+DSG-DpnII and FA+DSG-MNase(gray bars, right side), 2) number of overlapping loops detected (black bars). Overlapping loops are connected with black dots. **b**. Pileups of the loops from Fig. 4a. Numbers represent signal enrichment over local background (Methods). **c**. Individual loop strength (as in panel b) between protocol pairs in HFFc6. Protocols (left to right): FA-DpnII v/s FA+DSG-DpnII, FA+DSG-DpnII v/s FA+DSG-MNase and FA-DpnII v/s FA+DSG-MNase. Plots display two sets of looping interactions - Union (red squares) and Intersection (blue circles) from the three protocols. Color scale represents density of loop interactions. d,e.Aggregated loop strengths of intersection loop set from matrix of 12 protocols (described in Fig. 1a) for H1-hESC (d), and HFFc6 (e). Source data

First, we compared the number of loops detected in individual and merged biological replicates for the deeply sequenced protocols. We observed that the number of loops detected with protocols that cross-link chromatin with only FA was more sensitive to sequencing depth and less consistent between replicates compared with the number of loops detected using protocols that cross-linked with FA + DSG (Extended Data Fig. 4c,d). We used the lists of loops that were detected in merged replicates for subsequent analyses. In H1-hESCs we detected 3,951 loops with the FA–DpnII protocol, 12,396 loops with the FA + DSG–DpnII protocol, and 22,507 loops with the FA + DSG–MNase protocol (Extended Data Fig. 5a). For HFFc6 these numbers were 13,867, 22,934 and 36,988, respectively (Fig. 4a). To investigate the properties of detected loops, we compared loops that were called in individual or multiple protocols. Although a large fraction of loops was detected by all three protocols, we found that the protocols with extra cross-linking and finer fragmentation (FA + DSG–MNase) detected a large set of additional loops (Fig. 4a).

When we aggregated the interaction data for the various subsets of loops, we observed a focal increase in interaction frequency for all subsets of loops for all datasets, even for data obtained with protocols in which that subset of loops was not detected as significantly enriched (Fig. 4b for HFFc6 cells and Extended Data Fig. 5b for H1-ESCs). Quantifying the strength of the different subsets of loops detected by one or multiple protocols, we found that loops detected by all three protocols were the strongest, while loops detected only by the FA + DSG–MNase protocol were relatively weak.

We then defined a consensus set of loops that were detected in all datasets and used this set to analyze the data obtained with the matrix of 12 protocols described in Fig. 1 that differ in cross-linking and fragmentation strategies. We observed a gradual increase in average loop strength with decreasing fragment size and after addition of DSG or EGS (Extended Data Fig. 5d,e).

To explore this in another way, we quantified the strengths of each loop in the sets of consensus and union loops and found that the majority of loops were strengthened by additional DSG cross-linking (Fig. 4c, left panel) and by digestion with MNase as compared with DpnII (Fig. 4c, middle). Loops were strongest when additional cross-linkers and fragmentation with MNase were applied (Fig. 4c, right plot). A similar trend is also observed in HFFc6 cells (Extended Data Fig. 5c). We conclude that the use of additional cross-linkers and enzymes that fragment chromatin into smaller fragments independently contribute to the loop detection and strength.

**Fig. 5:**
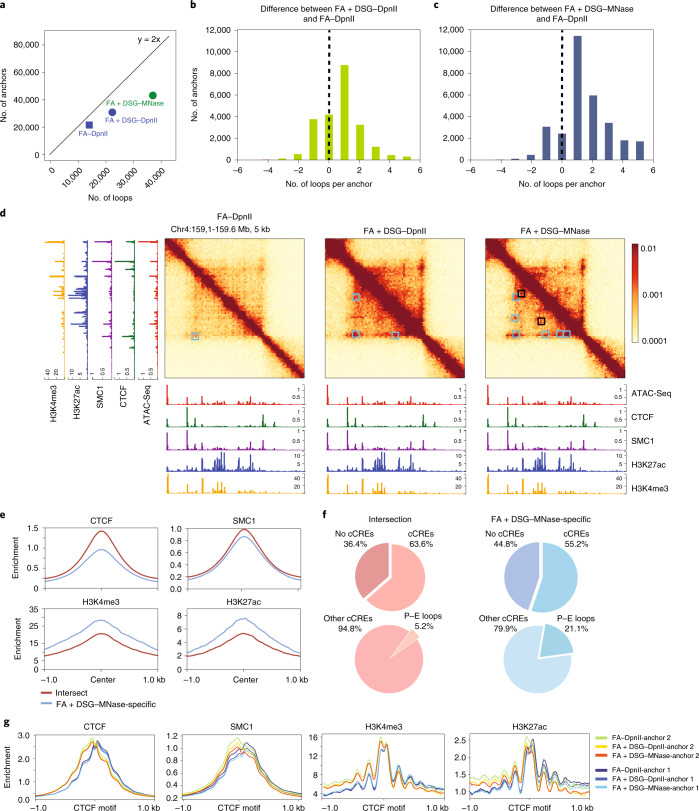
Characterization of interactions and chromatin features of loop anchors. **a**, The number of loops versus the number of loop anchors in HFFc6 cells, and the expected relationship between anchors engaged in one loop: y = 2x. **b**,**c**, Subtraction of FA–DpnII loops from FA + DSG–DpnII loops (**b**) or from FA + DSG–MNase loops (**c**) detected at the same anchors. Union loops of the plotted protocols were used. **d**, Chromatin interaction maps (linear scale) flanked by tracks for ATAC-seq and CUT&RUN or CUT&Tag signals for CTCF, SMC1, H3K4me3 and H3K27ac. Squares in the interaction maps indicate loop anchors detected with all three protocols (cyan squares) or only with the FA + DSG–MNase protocol (black squares)^32^. **e**, CTCF, SMC1, H3K4me3 and H3K27ac enrichments at loop anchors detected by all protocols (intersection) or by FA + DSG–MNase alone in HFFc6 cells. Open chromatin regions within anchor coordinates were used to center average enrichments. **f**, cCREs detected in common and in FA + DSG–MNase-specific loop anchors in **e** (top) and stratified percentage of promoter–enhancer (P–E) cCREs without CTCF enrichment (bottom). **g**, Enrichment of CTCF, SMC1, H3K4me3 and H3K27ac at the left (anchor 1) and the right (anchor 2) anchors, for anchors detected in HFFc6 cells using FA–DpnII, FA + DSG–DpnII or FA + DSG–MNase. Source data

**Extended Data Fig. 6:**
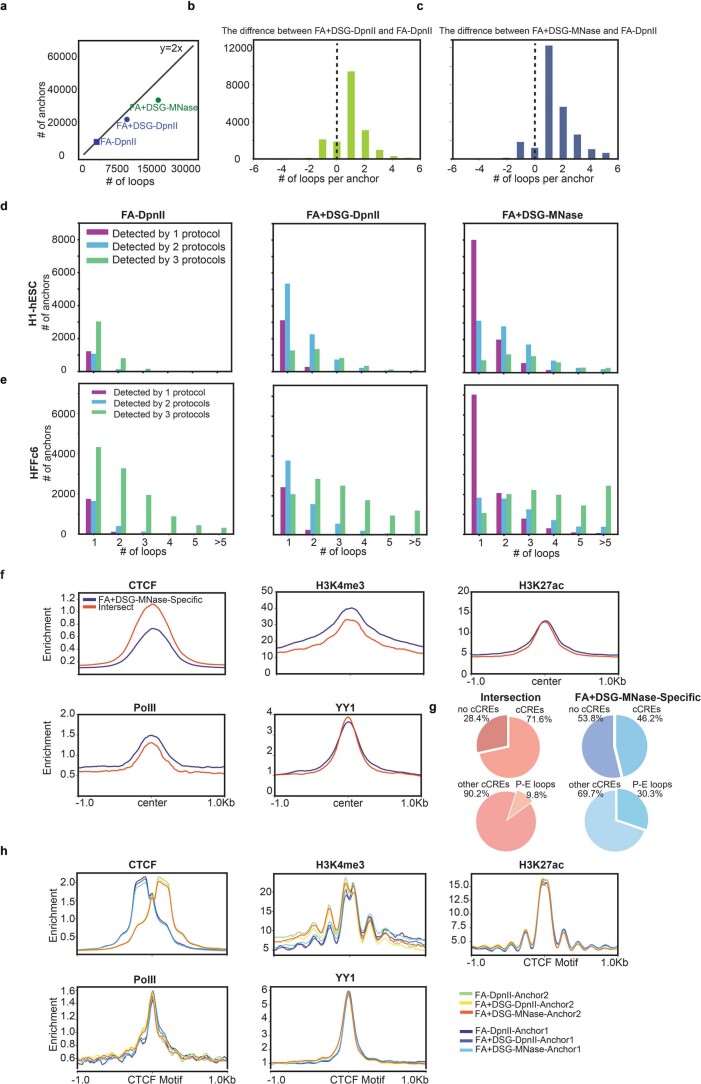
Finer fragments lead to detection of Promoter-Enhancer loops. **a**. H1-hESC loops versus loop anchors. Expected for anchors in one loop: y=2x **b,c**. FA-DpnII loops subtracted from FA+DSG-DpnII (b) or FA+DSG-MNase (c) Union of loops detected at the same anchors. **d,e**. Valencies of loop anchors for H1-hESC (d), HFFc6 (e) from FA-DpnII, FA+DSG-DpnII, FA+DSG-MNase. FA-DpnII is used as a guiding example. Categories are: anchors from 1 protocol (FA-DpnII), anchors from 2 protocols (FA-DpnII and either FA+DSG-DpnII or FA+DSG-MNase) and anchors from all 3 protocols. **f**. CTCF, SMC1, H3K4me3 and H3K27ac enrichments at loop anchors for all protocols (intersection) or FA+DSG-MNase alone in H1-hESC.Average enrichments centered on open chromatin regions within anchor coordinates. **g**. Top: cCREs from common and FA+DSG-MNase specific loop anchors (Extended Data Fig. 5f). Bottom: stratified percentage of Promoter-Enhancer cCREs without CTCF enrichment. **h**. Enrichment of CTCF, SMC1, H3K4me3 and H3K27ac for left (Anchor1) and right (Anchor 2) anchor in H1-hESC using FA-DpnII, FA+DSG-DpnII or FA+DSG-MNase. Source data

A looping interaction is defined by a pair of frequently interacting loci or anchors. When anchors engage in multiple looping interactions with other anchors, the number of anchors will be smaller than twofold the number of loops^5^. We compared the number of anchors as a function of the number of loops detected in deeply sequenced datasets for HFFc6 cells (Fig. 5a). We found that they were proportional to each other at a factor of 2 in the FA–DpnII experiment, but not in the experiments with improved loop detection (FA + DSG–DpnII and FA + DSG–MNase). This suggests that many of the newly identified loops involved anchors that were also detected with FA–DpnII (Fig. 5a and Extended Data Fig. 6a). In other words, many additionally detected loops are arranged along stripes emanating from the same anchors.

To further investigate this we directly determined the number of loops that a given anchor is engaged in as detected by different protocols. For each anchor, we subtracted the number of loops detected using the FA–DpnII protocol from the number of loops detected using the FA + DSG–DpnII or the FA + DSG–MNase protocol. We found that using extra cross-linkers as well as finer fragmentation increased the number of detectable loops (Fig. 5b,c and Extended Data Fig. 6b,c) in two ways: first, more loops are detected per anchor, and second, additional anchors are detected.

We split loop anchors into two categories: anchors detected with more than one protocol, and anchors detected with only one protocol. We observed that anchors detected with at least two protocols were engaged in multiple loops (loop ‘valency’ > 1). In contrast, anchors that were detected with only one protocol mostly had a loop valency of 1 (Extended Data Fig. 6d,e). Interestingly, for H1-hESCs the majority of additional loops detected with the FA + DSG–MNase protocol (62%) involve two anchors not detected with other protocols. For HFFc6 cells this was only 21%, indicating that most new loops shared at least one anchor with loops detected with other protocols.

We investigated factor binding (CTCF and cohesin (SMC1), YY1 and RNA PolII) and chromatin state (H3K4me3, H3K27ac) at the two categories of loop anchors. We used previously published datasets^20,21^ and new data generated using a variety of techniques (CUT&RUN^22^, CUT&Tag^23^, ChIP-Seq and ATAC-Seq^24^). Some loop anchors were detected with all protocols, and in the example shown these correspond to sites bound by CTCF and cohesin (Fig. 5d). Other loop anchors that were detected only with the FA + DSG–MNase protocol did not correspond to CTCF and cohesin-bound sites, but were enriched in H3K27ac and H3K4me3 (Fig. 5d). Possibly, the ability of different protocols to detect various loop anchors is related to factor binding and chromatin state. To investigate this across the whole genome we aggregated CTCF, SMC1, YY1 and RNA PolII binding data and histone modification data (H3K4me3 and H3K27ac) at loop anchors detected with all protocols or with only FA + DSG–MNase (Fig. 5e). Interestingly, in HFFc6 cells we found that FA + DSG–MNase-specific loop anchors were less enriched for CTCF and SMC1 but were more enriched for H3K4me3 and H3K27ac compared with the loop anchors that were detected by all three protocols (Fig. 5e and Extended Data Fig. 6f).

Next, we examined the predicted candidate *cis*-regulatory elements (cCREs) that are located at shared loop anchors across the three deep datasets and at loop anchors detected only with the FA + DSG–MNase protocol. We used cCRE predictions from the Encyclopedia of DNA Elements (ENCODE) for H1-hESCs and HFFc6 cells^25^. We found that the majority of the shared anchors had cCREs but only a small proportion of these cCREs were predicted promoter or enhancer elements without a CTCF site (5.2% for HFFc6 cells, 9.8% for H1-ESCs; Fig. 5f and Extended Data Fig. 6g). In contrast, half of the FA + DSG–MNase-specific anchors had predicted cCREs and for this subset the number of predicted promoter or enhancer elements without a CTCF site is higher compared with loop anchors detected with all protocols (21% for HFFc6 cells, 30% for H1-ESCs; Fig. 5f and Extended Data Fig. 6g). The FA + DSG–DpnII-specific loop anchors show similar enrichments to the FA + DSG–MNase-specific anchors.

Finally, we compared the chromatin organization at CTCF-enriched loop anchors with respect to the orientation of the CTCF-binding motif. Remarkably, using CUT&Tag or CUT&RUN data we found an asymmetric distribution of signal for all factors (Fig. 5g), including CTCF (CUT&Tag data). Both CTCF and cohesin signals were skewed towards the inside of the loop. We noted that the CUT&Tag data were generated with an antibody against the N terminus of CTCF (Fig. 5g). We also analyzed CUT&RUN data that were generated with an antibody directed against the C terminus of CTCF (Extended Data Fig. 6h) and observed signal enrichment skewed at CTCF sites towards the outside of the loop. These observations are consistent with the orientation of CTCF binding to its motif and interactions between the N terminus of CTCF with cohesin on the inside of the loop^26^. The stronger enrichment of H3K4me3 and H3K27ac on the inside of the loop is intriguing, but the mechanism of this asymmetry is still unknown.

### Insulation quantification is robust to experimental variations

Next we investigated chromatin insulation, that is, the reduced interaction probability across domain boundaries^27–29^. Loop anchors often form domain boundaries given that they represent sites at which cohesin-mediated loop extrusion is blocked. To identify sites of insulation using the previously described insulation metric^30^, we performed three separate analyses.

**Extended Data Fig. 7:**
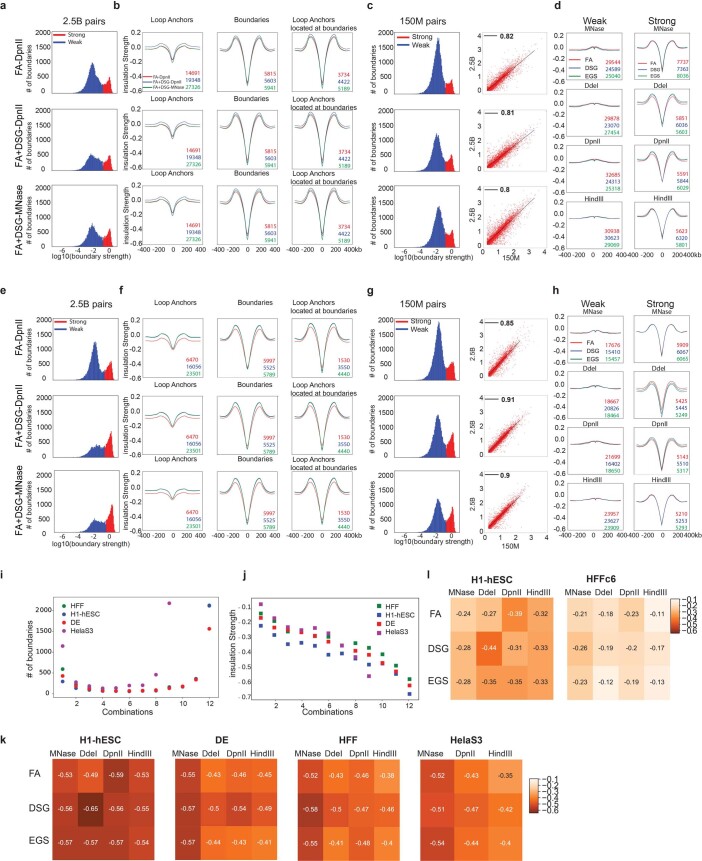
Insulation quantification is robust to experimental variations. **a-d**. Rows: HFFc6 deep data from FA-DpnII (top), FA+DSG-DpnII (middle) and FA+DSG-MNase (bottom). Columns: boundary strength distributions with strength threshold (a) (Methods), pileups in FA-DpnII, FA+DSG-DpnII and FA+DSG-MNase for aggregate insulation scores at loop anchors (left), strong insulation boundaries (middle) and loop anchors colocalizing with strong insulation boundaries (right) (b). Left panel: boundary strength distribution of matrix data (Fig. 1a) for FA-DpnII, FA+DSG-DpnII and FA+DSG-MNase. Right panel:correlation of strong boundaries between deep and matrix data for FA-DpnII, FA+DSG-DpnII and FA+DSG-MNase (c). Aggregate insulation profile of weak and strong boundaries from matrix data for HFFc6 for cross-linkers and nucleases (d). **e–h**. H1-hESC data displayed like Extended Data Fig. 7a-d. **i**. Number of boundaries (y-axis) stratified by number of protocols (1 to 12; see Fig. 1a) wherein a given boundary was detected (x-axis). **j**. Insulation strength of boundaries stratified as in (i). **k**. Mean insulation strength for boundaries detected in at least half of protocols for various cross-linkers and enzyme combinations of H1-hESC, DE, HFF and HeLa-S3-NS (Methods). **l**. Mean insulation strength of loop anchors detected in all three deep protocols for both HFFc6 and H1-hESC, averaged for 12 protocols of H1-hESC and HFF. Source data

First, we compared the boundary strength as detected with the deep datasets obtained with the FA–DpnII, FA + DSG–DpnII and FA + DSG–MNase protocols in HFFc6 cells. The distribution of the boundary strengths was bimodal: for each dataset we identified a relatively large set of very weak boundaries, and a smaller set of strong boundaries (Extended Data Fig. 7a). Insulation at the weak boundaries was very small, and was possibly due to noise (Extended Data Fig. 7d). Focusing on the strong boundaries, we aggregated insulation profiles at three points: loop anchors detected with each of the three deep datasets; strong boundaries; and loop anchors at strong boundaries (Extended Data Fig. 7b). Insulation was very similar for each of the three deep datasets, indicating that the different protocols performed similarly in the quantitative detection of strong insulation sites. In general, insulation at strong boundaries was stronger than at loop anchors, possibly because of the stringent threshold for boundary detection.

Second, we investigated whether insulation strength depends on sequencing depth. We compared two biological replicates, one with ~150 million interactions (matrix data, Extended Data Fig. 7c) and the other with 2.5 billion interactions (deep data, Extended Data Fig. 7a) for data obtained with the FA–DpnII, FA + DSG–DpnII and FA + DSG–MNase protocols. Deeper sequencing reduced the relative number of weak boundaries, suggesting that these were due to noise. The majority (>85%) of strong boundaries are detected in both deep data and the less deeply sequenced data obtained with the matrix of 12 protocols, and the insulation scores of these shared strong boundaries were highly correlated across all datasets (r > 0.80) (Extended Data Fig. 7c).

Last, we investigated the number and the strength of the boundaries detected using data obtained with the matrix of 12 protocols for HFF cells, H1-hESCs, DE cells and the 9 protocols for HeLa-S3 cells. Insulation strength at boundaries detected with each protocol was very similar (Extended Data Fig. 7d). We observed the same results for H1-hESCs (Extended Data Fig. 7e–h).

We found a positive correlation between boundary strength and the number of protocols that detected that boundary (Extended Data Fig. 7i,j). Focusing on the set of boundaries that were detected by at least half of the protocols, we then investigated how insulation varied for data obtained with the matrix of 12 protocols. We found that insulation strength was very similar for data obtained with all of the protocols (Extended Data Fig. 7k). Similarly, we detected only minor variations in insulation when insulation was aggregated at the set of loop anchors detected by all three deep datasets using data obtained with the matrix of 12 protocols. In summary, insulation detection and quantification was robust to variations in protocol (Extended Data Fig. 7l).

### Hi-C 3.0 detects both compartments and loops

**Extended Data Fig. 8:**
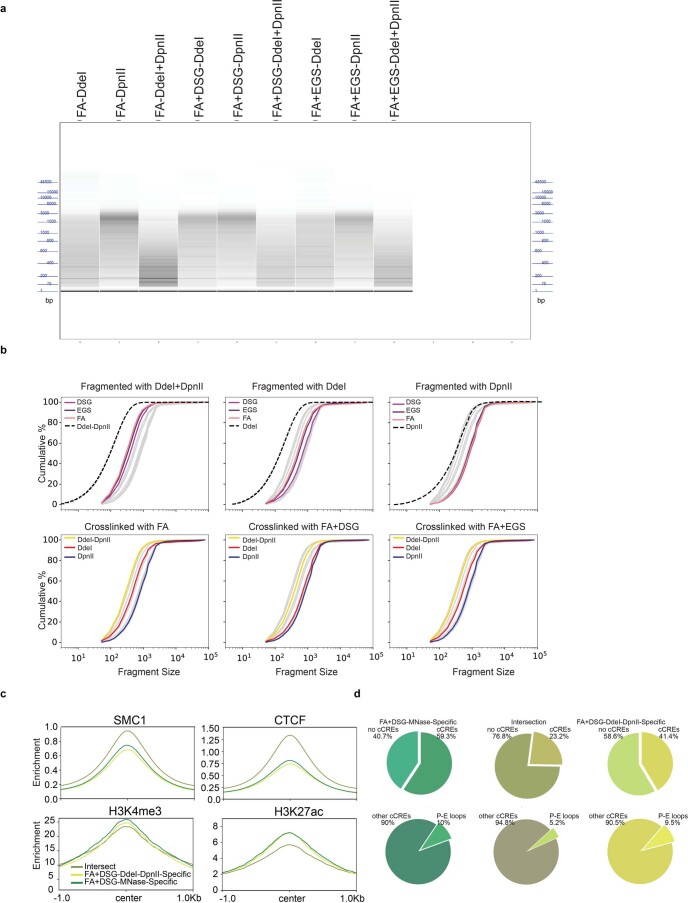
Hi-C 3.0 performs similar to Micro-C. **a**. Fragment size distributions from Fragment Analyzer for specified protocols. **b**. Cumulative distributions of fragmented DNA in HFF cells stratified for cross-linking agents (top row) or restriction enzymes (bottom row). Dashed lines in each of the panels represent expected fragment size distribution from *in silico* digestion of hg38 for enzymes indicated. Gray lines represent all data from all other enzymes (columns). **c**. Comparison of CTCF, SMC1, H3K4me3 and H3K27ac enrichments at loop anchors centered at open chromatin regions. Open chromatin regions (ATAC Seq) located within the anchor coordinates were used to center the average enrichments. Anchors were separated into sets detected by FA+DSG-DdeI+DpnII, FA+DSG-MNase or both. **d**. Percentage of cCREs and promoter-enhancer elements located at loop anchors specific to FA+DSG-DdeI+DpnII, FA+DSG-MNase or shared between them. Source data

We showed that additional cross-linkers strengthen the compartment signal and loop enrichments. Additionally, compartments were strongest for experiments that have longer fragments, and loops were better detected when the chromatin was fragmented into smaller fragments. We considered whether a single protocol could be designed to optimally capture both compartments and loops. We tested the effect of digestion with both DdeI and DpnII after cross-linking with FA + DSG (FA + DSG–DdeI + DpnII, referred to as ‘Hi-C 3.0’). We observed that using two enzymes further shortened the fragment size compared with individual enzyme digestion (Extended Data Fig. 8a,b). Applying this protocol to HFFc6 cells, we generated two deeply sequenced biological replicates (3.3 billion valid interactions combined). For comparison, we also generated a dataset using only DdeI digestion (FA + DSG–DdeI; 2.7 billion valid interactions) in addition to the deeply sequenced libraries digested with only DpnII or MNase described above.

**Fig. 6:**
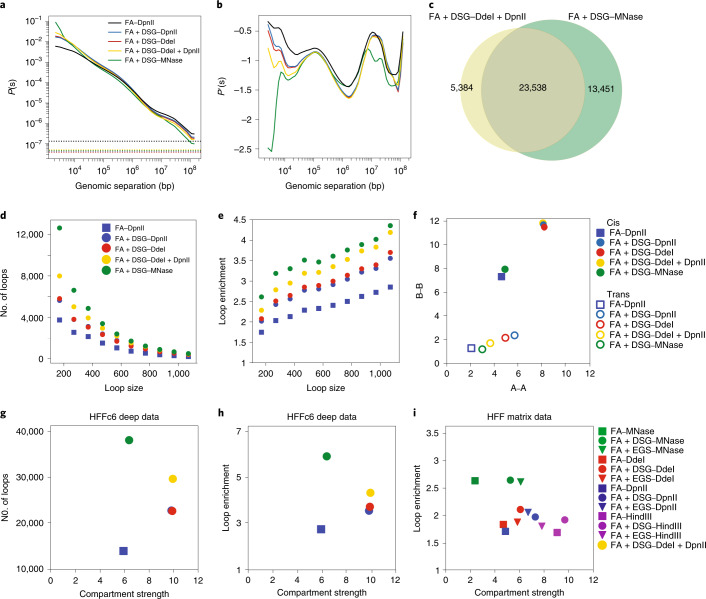
Hi-C 3.0 detects both compartments and loops. **a**, Distance-dependent contact probability of interactions detected with five protocols in HFFc6 cells (mean of trans interactions is shown in dashed lines). **b**, Derivative of the *P*(s) plots from **a**. **c**, Overlapping loops between FA + DSG–DdeI + DpnII and FA + DSG–MNase. **d**, Number of loops detected in 100-kb intervals (loop size) starting at 70 kb. **e**, Loop enrichment of 1,000 loops sampled from 100-kb intervals in **d**. When less than 1,000 loops were available, loop strengths for available loops were used. **f**, A–A and B–B compartment strengths in cis and in trans derived from saddle plot analysis. **g**, Number of loops and compartment strength for five protocols applied to HFFc6 cells. **h**, Compartment strength compared with loop enrichment for 10,000 loops sampled from HFFc6 cells (2,000 loops were sampled from each protocol). **i**, Compartment strength for 12 protocols described in Fig. 1a is compared to loop enrichment of these protocols using 10,000 sampled loops (in **h**) using data from the same 12 protocols. Source data

We found that the FA + DSG–DdeI + DpnII protocol affected the distance-dependent contact probability (Fig. 6a). Compared with data obtained by single DdeI or DpnII digests, in the data obtained with the FA + DSG–DdeI + DpnII protocol the contacts increased for loci separated by less than 10 kb, making the results from this protocol more similar to results obtained with protocols using MNase digestion. However, longer distance contacts more closely resembled data obtained with protocols using single restriction enzymes than data obtained with protocols using MNase. Combined, this protocol improved the short-range signal without loss of the long-range signal (Fig. 6b).

We found that the majority of the loops detected with FA + DSG–DdeI + DpnII overlapped with those detected with FA + DSG–MNase (Fig. 6c). Loop anchors detected in both protocols had stronger CTCF and cohesin enrichment, whereas protocol-specific anchors (for both FA + DSG–DdeI + DpnII and FA + DSG–MNase) were more enriched for H3K4me3 and H3K27ac (Extended Data Fig. 8c). Loop strength increased compared with data obtained with protocols that use a single restriction enzyme. We found ~6,000 more looping interactions than with either single DpnII or single DdeI digestion (Fig. 6d). Furthermore, the average enrichment of contacts at these looping interactions was also higher for data obtained with the double digestion protocol. Nonetheless, the MNase library remained superior in detecting loops, both in number and in contact enrichment (Fig. 6e). Importantly, when we investigated compartmental interactions we found that smaller restriction fragments did not result in a loss of quantitative detection of preferential compartmental interactions (Fig. 6f). In addition, the FA + DSG–DdeI + DpnII double-digest protocol allows for the efficient detection of both loops and compartments in a single protocol (Fig. 6g,h).

**Extended Data Fig. 9:**
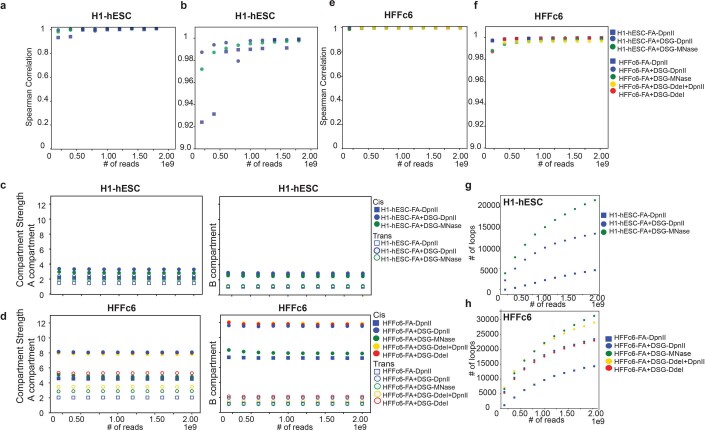
Sequencing depth impact loop detection but not compartmentalization. **a**. Spearman correlation of the eigenvectors for different sequencing depths in H1-hESC. Each point represents one sampled experiment. X-axis shows the sequencing depth (200 M reads-2B reads) and the y- axis shows the correlation of the eigenvectors for each depth with the eigenvector of the experiment with 2 Billion reads. The bottom plot shows the zoomed correlations. **b**. Compartment strength of A compartment for experiments with different read depths quantified in cis and trans for H1-hESC. **c**. Compartment strength of B compartment for experiments with different read depths quantified in cis and trans for H1-hESC. **d**. # of loops detected in experiments with different read depths in H1-hESC. **e-f**. Analysis that is shown in a-d repeated for experiments performed in HFFc6 cells. Source data

Finally, we tested how compartment strength and loop detection changes for various sequencing depths. We compared experiments from H1-hESC and HFFc6 deep datasets and sampled ten times, resulting in a range from 200 million to 2 billion reads. First, we found that compartment identifications are similar for all read depths (Spearman correlation > 0.9) (Extended Data Fig. 9a,b). Second, we observed that the compartment strength does not change for different read depths (Extended Data Fig. 9c). And last, more loops are detected as the number of reads increases (Extended Data Fig. 9d). Importantly, at all read depths the number of detected loops increases with finer fragmentation and additional cross-linking.

## Discussion

We observed that fragmentation level and cross-linking chemistry influenced detection of chromatin loops and compartmentalization. Loop detection was improved when chromatin was cross-linked with additional (DSG) cross-linking and cut into small fragments. Loops detected with such protocols were more enriched for cis elements such as enhancers and promoters as compared with sets of loops detected with the conventional Hi-C protocol. However, this comes at the cost of a reduced ability to quantitatively detect compartmentalization in cis and in trans. Quantification of compartmentalization improved with longer fragments such as those produced with DpnII in the conventional Hi-C protocol. Compartment strength improved with additional cross-linkers or when chromatin was digested with HindIII. We showed that Hi-C 3.0 using two restriction enzymes (DpnII and DdeI) and additional DSG cross-linking combined the strengths of the MNase-based Micro-C protocol to detect loops and the Hi-C protocols in detecting stronger compartments.

Fragmentation level and cross-linking chemistry determine assay performance by affecting the level of noise due to random ligation events in datasets^14^. We find that smaller fragments result in more random ligation events, possibly due to the low number of cross-links per fragment for small fragments, leading to a higher mobility and increased random ligations during the assay. Random ligation events diminish when additional cross-linking is used or when chromatin is fragmented into larger fragments. This results in a decrease in inter-chromosomal interactions and steeper *P*(*s*) plots. Improved signal-to-noise ratios allowed better detection of loops, compartments and more bona fide inter-chromosomal interactions.

Detection of compartmentalization strength is improved when protocols are used that produce relatively long fragments and include additional cross-linking. Possibly, compartmental interactions are more difficult to capture than looping interactions that are closely held together by cohesin complexes. Recently, we found that interfaces between compartment domains appear relatively unmixed^31^. Longer fragments or extra cross-linkers may be required to more efficiently capture contacts across these interfaces. Interestingly, cell type-specific differences in strength of compartmentalization are observed only with some protocols. Conventional Hi-C (FA + DpnII) suggests that compartmentalization strength is quite similar in H1-ESCs, HeLa-S3 cells, DE cells and HFF cells. However, when Hi-C is performed with additional cross-linkers and/or with restriction enzymes that produce longer fragments, HFF and HeLa-S3 cells have stronger compartmentalization, while the compartmentalization strength for H1-ESCs and DE cells are unaffected. This suggests that quantitative differences in cell type-specific chromosome organization can be missed or underestimated depending on the 3C-based protocol.

Insights into the influence of experimental parameters of chromatin interaction data led us to test a single Hi-C protocol (Hi-C 3.0), that can be used for better detection of both loops and compartments. Hi-C 3.0 produces shorter fragments than those in conventional Hi-C, but not as short as in the nucleosome-sized fragments in Micro-C. Hi-C 3.0 allows detection of thousands more loops compared with conventional Hi-C, and stronger compartmentalization than Micro-C. Depending on the objective of the study, investigators may choose different protocols: Micro-C for loop detection, or Hi-C 3.0 for detection of both loops and compartments. Hi-C 3.0 may be a good compromise protocol for many studies. Finally, we recommend always using FA + DSG (or EGS) cross-linking.

The deeply sequenced Hi-C, Micro-C and Hi-C 3.0 datasets we produced for H1-ESCs and HFFc6 cells will be useful resources for the chromosome folding community given that these cell lines are widely used for method benchmarking and analysis by the 4D Nucleome project^19^. Furthermore, the comprehensive collection of chromatin interaction data generated with the matrix of the 12 3C-based protocol variants for each cell line can also be a valuable resource for benchmarking computational methods for data analysis, given their different cross-linking distances and chemistry, fragment lengths and noise levels.

## Methods

### cLIMS: a laboratory information management system for C-data

cLims is a web-based lab information management system tailored to 3C experiments. It can be used to organize, store and export metadata of various experiment types such as Hi-C, 5C, ATAC-Seq and so on. The metadata organization is compatible with 4D Nucleome (4DN) Data Coordination and Integration Center (DCIC) standards, and cLIMS can be used to export data to 4DN DCIC and Gene Expression Omnibus (GEO) systems with one click.

For the matrix project, we had increasing levels of detail in metadata, a growing number of experiments, long time periods between data creation and submission and many people working on the same datasets, hence cLIMS enabled the information to be properly maintained. The details included cell lines, assays, treatments, sequencing and contributor information. This will also facilitate experiment reproducibility.

cLIMS was developed using the Django web framework on the back end and HTML5 and Javascript libraries on the front end. It is run on a PostgreSQL database and Apache web server and can be hosted on major Linux distributions.

### Cell line culture and fixation

#### HFFc6 cells

HFFc6 cells were cultured according to 4DN SOP (https://data.4dnucleome.org/biosources/4DNSRC6ZVYVP/). Cells were grown at 37 °C under 5% CO_2_ in 75 cm^2^ flasks containing DMEM, supplemented with 20% heat-inactivated FBS. For sub-culture, cells were rinsed with 1× DPBS and detached using 0.05% trypsin at 37 °C for 2–3 min. Cells were typically split every 2–3 d at a 1:4 ratio and collected while sub-confluent, to ensure that they would not overgrow.

#### H1-hESC

The hESCs (H1, WiCell, WA01, lot no. WB35186) were cultured in mTeSR1 media (StemCell Technologies, 85850) under feeder-free conditions on Matrigel H1-hESC-qualified matrix (Corning, 354277, lot no. 6011002)-coated plates at 37 °C and 5% CO_2_. H1 cells were fed daily with fresh mTeSR1 media and passaged every 4–5 d using ReLeSR reagent (StemCell Technologies, 05872). Cells were dissociated into single cells with TrypLE Express (Thermo Fisher, 12604013).

#### Fixation protocol

The final collection of 5 million HFFc6 and H1-hESC cells was performed after washing twice with Hank’s Buffered Salt Solution (HBSS) before cross-linking in HBSS with 1% FA for 10 min at room temperature. FA was quenched with glycine (128 mM final concentration) at room temperature for 5 min and on ice for an additional 15 min. Cells were washed twice with DPBS before pelleting and flash freezing with liquid nitrogen into 5 million aliquots. Alternatively, FA-fixed cells were centrifuged at 800×*g* and subjected to additional cross-linking with either 3 mM DSG or EGS, freshly prepared and diluted from a 300 mM stock in DMSO, for 40 min at room temperature. DSG and EGS cross-linked cells were both quenched with 0.4 M glycine for 5 min and washed twice with DPBS, supplemented with 0.05% BSA, before flash freezing with liquid nitrogen into 5 million aliquots.

#### Hi-C protocol

Chromosome conformation capture was performed as described previously and we refer to Belaghzal et al.^33^ for a step-by-step version similar to this protocol. The optimizations of cross-linkers are described above.

#### Micro-C-XL protocol

The Micro-C XL protocol was adopted from Hsieh et al. and Krietenstein et al.^7,8^.

#### Size range of chromatin fragments produced after digestion

Cells were cross-linked, lysed and digested as with the Hi-C protocol (see above). Then, cross-links were reversed and DNA was isolated as in Hi-C, but without ligation and biotin incorporation. DNA was loaded on an Advanced Analytical Fragment Analyzer (Agilent) for size range analysis, and the data were analyzed using PROsize3 software (Agilent). PROsize3 traces were exported separately as 4 × 8 bins (32 total) in ranges of 40–500, 500–1,300, 1,300–8,000 and 8,000–100,000 bp. Size ranges of potential restriction sites (hg38) were identified using cooltools genome digest (https://cooltools.readthedocs.io/en/latest/cli.html?highlight=enzyme#cooltools-genome-digest).

#### CUT&Tag protocol

Samples were processed as previously described^23^ with few modifications. In brief, approximately 100,000 cells per sample were permeabilized in the wash buffer (20 mM HEPES pH 7.5, 150 mM NaCl, 0.5 mM Spermidine, 1× Protease inhibitor cocktail), and then cells were coupled with activated concanavalin A-coated magnetic beads for 10 min at room temperature. Pelleted beads were resuspended in antibody buffer (Mix 8 μl 0.5 M EDTA and 6.7 µl 30% BSA with 2 ml Dig-wash buffer) with 1:100 dilution of SMC1 (Bethyl, cat. no. A300-055A) or CTCF antibody (Active motif, cat. no. 61311) and incubated overnight at 4 °C on a rotator. The next day, the pelleted bead complex was incubated with 1:50 dilution of secondary antibody (guinea pig α-rabbit antibody, cat. no. ABIN101961) in Dig-Wash buffer (20 mM HEPES pH 7.5, 150 mM NaCl, 0.5 mM Spermidine, 1× Protease inhibitor cocktail, 0.05% Digitonin) and incubated at room temperature for 30 min on a rotator. After two washes in Dig-Wash buffer, 1:250 diluted pAG-Tn5 adapter complex in Dig-300 buffer (20 mM HEPES pH 7.5, 300 mM NaCl, 0.5 mM Spermidine, 1× Protease inhibitor cocktail, 0.05% Digitonin) were added to the bead complex and incubated at room temperature for 1 h. After two washes in Dig-300 buffer, beads were resuspended in 300 µl Tagmentation buffer (20 mM HEPES pH 7.5, 300 mM NaCl, 0.5 mM Spermidine, 1× Protease inhibitor cocktail, 0.05% Digitonin, 10 mM MgCl_2_) and incubated at 37 °C for 1 h 45 min. Samples underwent Proteinase K treatment and extraction of tagmented DNA using phenol : chloroform : isoamyl alcohol (25:24:1). In preparation for Illumina sequencing, 21 µl DNA was mixed with 2 µl universal i5, 2 µl uniquely barcoded i7 primer, and 25 µl NEBNext High-Fidelity 2X PCR Master mix. The sample was placed in a thermocycler with a heated lid using the following cycling conditions: 72 °C for 5 min, 98 °C for 30 s, 14 cycles of 98 °C for 10 s and 63 °C for 30 s, with a final extension at 72 °C for 1 min and hold at 4 °C. Post-polymerase chain reaction (PCR) clean-up was performed by adding 1.1× volume Ampure XP beads and incubating for 15 min at room temperature, washing twice gently in 80% ethanol, and eluting in 30 µl 10 mM Tris pH 8.0. The final library samples were paired-end sequenced on Nextseq500.

#### CUT&RUN protocol

CUT&RUN raw data (fastq files) of H1-hESC are downloaded from Janssens et al.^20^ and raw files of HFFc6 are generated in the laboratory of S. Henikoff using the protocol developed by Skene and Henikoff^22^.

#### ATAC-Seq protocol

We have followed a published protocol to perform H1-hESC ATAC-Seq experiments. The protocol details have been described previously.^34^

ATAC-Seq experiments on HFFc6 cells were performed following a previously published protocol.^24^ In brief, 50,000 cells per experiment were washed and lysed using a lysis buffer (0.1% NP-40, 10 mM Tris-HCl pH 7.4, 10 mM NaCl and 3 mM MgCl_2_). Lysed cells were then transposed using the Nextera DNA library prep kit (Illumina, FC-121–1030) for 30 min at 37 °C, immediately followed by DNA collection using Qiagen MinElute columns (Qiagen, 28004). Appropriate cycle numbers for amplification were determined for each sample individually using quantitative PCR. Finally, primers were removed using AMpure XP beads (Beckman Coulter, A63881) prior to 2 × 50-bp paired-end sequencing.

### Data analysis

#### Chromosome capture data processing

The Distiller pipeline (https://github.com/mirnylab/distiller-nf) is used to process Hi-C and Micro-C datasets. First, sequencing reads were mapped to hg38 using bwa mem with flags-SP. Second, mapped reads were parsed and classified using the pairtools package (https://github.com/mirnylab/pairtools) to produce 4DN-compliant pairs files. We removed PCR and optical duplicates using the positions of aligned reads with 2 bp flexibility. Next, pairs were filtered using mapping quality scores (MAPQ > 30) on each side of aligned chimeric reads, binned into multiple resolutions, and low-coverage bins were removed. Finally multi-resolution cooler files were created using the cooler package^10^ (https://github.com/mirnylab/cooler.git). We normalized contact matrices using the iterative correction procedure from Imakaev et al.^11^. Interaction heatmaps were created using the ‘cooler show’ command from the cooler package.

#### HiCRep correlations

We used HiCRep to do distance-corrected correlations^12^ of the various protocols and cell states. Correlation is calculated in two steps. First, interaction maps are stratified by genomic distances and the correlation coefficients are calculated for each distance separately. Second, the reproducibility is determined by a novel stratum-adjusted correlation coefficient statistic (SCC) by aggregating stratum-specific correlation coefficients using a weighted average. We correlated 50-kb binned individual chromosomes between protocols and averaged the correlations across all chromosomes.

#### Cis and trans ratio

Trans percent is calculated by dividing the total interactions between chromosomes by the sum of the interactions within and between chromosomes (trans/cis + trans). Distance-separated cis interactions are calculated by dividing the total interactions within a specified distance of the chromosomes by the sum of interactions within and between the chromosomes (cis of specific distance/cis + trans). Pairtools provides statistics for the numbers of interactions captured within and between chromosomes.

#### *P*(s) plots

*P*(s) plots describe the decay of the average probability of contact between two regions on a chromosome as a function of the genomic separation between them.

As per best practice, scaling is typically computed for each chromosomal arm of the genome before being aggregated. To obtain the extent of each chromosomal ar the sizes of the chromosomes and the positions of their associated centromeres must be obtained. The sizes of the chromosome were obtained using the fetch_chromsizes function that is found in the bioframe library (https://github.com/open2c/bioframe/blob/master/bioframe/io/resources.py#L61), and the starts and ends of the centromere were obtained from bioframe using the function fetch_centromeres (https://github.com/open2c/bioframe/blob/master/bioframe/io/resources.py#L109). The results of these two functions were combined to create a single list containing the extents of each chromosomal arm of the human hg38 genome. For all libraries except those made from HeLa-S3 cells, all chromosome arms were used in the scaling calculation. For HeLa libraries we excluded the chromosomes with translocations and used only chromosomes 4, 14, 17, 18, 20 and 21.

We used the diagsum function from the cooltools library (https://github.com/open2c/cooltools/blob/master/cooltools/expected.py#L541) to calculate scaling. This function takes in a cooler, extracts the table of non-zero read-counts across the genome (known as the pixel table) and calculates the sum of read-counts as a function of distance from the main diagonal. It also simultaneously calculates the total number of possible counts obtainable at a given distance (called valid pairs) based on masking of the region due to balancing and other user-provided criteria. Additionally, this function can also transform the read-counts obtained from the pixel table before aggregating the result. This is done by passing the appropriate user-defined function to the ‘transforms’ parameter of diagsum.

To obtain the scaling plots shown in the paper, for each library, the diagsum function was applied on the 1-kb cooler associated with the library. The recommended resolution to calculate scalings is 1 kb because it allows us to observe variations at the finest scales. Along with the cooler, the extents of the chromosomal arms were also provided using the regions argument. A transform (named ‘balanced’) was also applied to the data to convert raw read-counts to balanced read-counts. This was done by multiplying the count value with the associated row and column weights obtained from balancing the cooler.

The resulting output is a single table with four relevant columns: ‘region’, which describes which chromosome arm a specific row was obtained from; ‘diag’, which refers to the genomic separation at which the data were aggregated; ‘balanced.sum’, which is the sum of read-counts for that given region and genomic separation after they were transformed by the balanced transform; and ‘n_valid’, which is the number of possible valid pairs at a given distance (as described earlier). The individual column values were aggregated over the different arms and then further aggregated into logarithmically spaced bins of genomic separation. Finally, the balanced.sum column was divided by the n_valid column to create the ‘balanced.avg’ column, which is a measure of the average number of contacts across the genome for a given genomic separation. The curves shown in the main text are the balanced.avg values plotted as a function of diag for the different libraries.

In addition to the interaction decay within a chromosome, interaction between different chromosomes can also be quantified. This is done using the blocksum_asymm function in cooltools (https://github.com/open2c/cooltools/blob/master/cooltools/expected.py#L820), which uses a very similar methodology. Two sets of regions are provided to blocksum_asymm, and then balanced.sum and n_valid are calculated for every pair of regions (entire chromosomes in this case). Given that the interactions are between two chromosomes there is no notion of genomic separation between two regions. Balanced.avg is calculated in the same manner as above and the mean of this value is visualized as horizontal dashed lines in the main text figures.

#### Average slope of scaling

To magnify small variations between the different libraries, we calculated derivative curves from the scaling curves. Derivative curves represent the rate of change of scaling curves as observed on a log–log scale. These are computed by taking the log of scaling data (both x and y), calculating the finite difference measure of the slope and then smoothing that value with a Gaussian kernel. The smoothing function used is gaussian_filter1d from the SciPy library (with a spread of 1). The smooth finite difference values can be plotted as a function of distance, as is the case for Fig. 6b. Alternatively, the average value of this derivative is calculated and correlated with other features (as in Fig. 2c,d).

#### Genome coverage analysis

For genome-wide coverage analysis, the mapped read pairs were split into two individual files and the read coverage at respective bins (genome-wide at 100-kb bins) was computed with the BEDTools coverage (v2.29.2) function. The read density was normalized to reads per million to compare between samples with different total read-counts and subsequently to reads per 1 kb to compare between annotations with different bin sizes. The compartment associations were extracted from HindIII compartment calls using the respective cell types.

#### Compartment analysis

We assessed compartments using eigenvector decomposition on observed-over-expected contact maps at a resolution of 100 kb separated for each chromosomal arm using the cooltools package-derived scripts. The eigenvector that has the strongest correlation with gene density is selected, then the A and B compartments were assigned based on the gene density profiles such that the A compartment has a high gene density and the B compartment has a low gene density profile^11^. Spearman correlation (Extended Data Fig. 3a) was used to correlate the eigenvectors of different experiments performed with various protocols and cell states. Saddle plots were generated as follows: the interaction matrix of an experiment was sorted based on the eigenvector values from lowest to highest (B to A). Sorted maps were then normalized for their expected interaction frequencies; the upper left corner of the interaction matrix represents the strongest B–B interactions, the lower right represents the strongest A–A interactions, and the upper right and lower left represent B–A and A–B, respectively. To quantify the saddle plots we took the strongest 20% of B–B interactions and the strongest 20% of A–A interactions, normalized by the bottom 20% of A–B interactions; that is, *y* = top(B–B)/bottom(A–B) and *x* = top(A–A)/bottom(A–B). Saddle quantification was used to create the scatter plots in Fig. 3c and heatmaps in Extended Data Fig. 3 that compare A and B compartments for all cell types. Both scatter plots and heatmaps in Fig. 3 and Extended Data Fig. 3 were created using the Matplotlib package from Python.

#### Identification of chromatin loops

The cooltools call-dots function (https://github.com/open2c/cooltools/blob/master/cooltools/cli/call_dots.py), a re-implementation of HICCUPS^3^, was used to detect the chromatin loops that are reflected as dots in the interaction matrix. We used the following parameters to call the loops: fdr = 0.1, diag_width = 10000000, tile_size = 5000000, and max-nans-tolerated 4. We called dots in deep data at resolutions of both 5 kb and 10 kb, using MAPQ > 30 pairs and merged the results using the criteria given by Rao et al.^3^ In brief, to merge 5-kb and 10-kb loop calls, both the reproducible 5-kb calls and the unique 10-kb calls were kept. Unique 5-kb calls were kept if the genomic separation of the region was <100 kb or if the dots were particularly strong (that is, more than 100 raw interactions per 5-kb pixel). More detailed explanations for dot calling are given by Rao et al. and Krietenstein et al.^3,8^.

#### Comparison of loops detected in different protocols

BEDTools intersect^35^ was re-implemented to overlap two-dimensional (2D) loops between protocols. Given that loop calls are fundamentally 2D data, they need to be processed for use with BEDTools (which operate on one-dimensional (1D) data).

Each loop call consists of six coordinates: chrom1, start1, end1, chrom2, start2 and end2. Given that chrom1 is always the same as chrom2 for loop calls, we ignored these two columns and reduced our space to four coordinates. Furthermore, to account for errors in the positioning of the loop during the loop calling, we introduced the following margin of error around the called region (typically 10 kb):

$$\begin{array}{l}{\mathrm{pos}}1 = ({\mathrm{start}}1 + {\mathrm{end}}1)/2;\quad {\mathrm{start}}1 = ({\mathrm{pos}}1 - 5\,{\mathrm{kb}});\quad {\mathrm{end}}1 = ({\mathrm{pos}}1 + 5\,{\mathrm{kb}})\\ {\mathrm{pos}}2 = ({\mathrm{start}}1 + {\mathrm{end}}1)/2;\quad {\mathrm{start}}2 = ({\mathrm{pos}}1 - 5\,{\mathrm{kb}});\quad {\mathrm{end}}2 = ({\mathrm{pos}}2 + 5\,{\mathrm{kb}}).\end{array}$$

To overlap the two lists, we performed two separate 1D overlaps with BEDTools and then merged the results. To this end, every entry on each list is given a unique loop identification. Using BEDTools overlap on each dimension of the loop list, we obtained a pair of loop identifications (one from each list) that were used to track which pairs of dots overlapped along both dimensions. Thus only pairs of dots with overlaps in both dimensions are merged and outputted.

#### Upset plots

Upset plots were created for overlapping loops using the following R package: https://cran.r-project.org/web/packages/UpSetR/vignettes/basic.usage.html.

### Quantification of chromatin loops

We created the loop pileups using notebooks from the hic-data-analysis-bootcamp notebook (https://github.com/hms-dbmi/hic-data-analysis-bootcamp/blob/master/notebooks/06_analysis_cooltools-snipping-pileups.ipynb). The pileups were done at a resolution of 5 kb and with a 50-kb extension on each side of the loop. To quantify the loop strength, first, we created an interaction matrix of 50 × 50 kb, centered around the loop. Then, we calculated the intensity of the loop by dividing the average of a 3 × 3 square in the middle of the interaction matrix by the average of its neighboring pixels: upper left, upper middle, upper right; middle right; and lower right (Supplementary Fig. 1).

This quantification of loop enrichment using its local background was also done to identify the loops. These quantifications are shown in Fig. 4b,c and Extended Data Fig. 5b–e.

#### Anchor analysis

We concatenated the genomic positions of the left and the right anchors for each loop to create a 1D anchor list for each deep dataset (FA–DpnII, FA + DSG–DpnII, FA + DSG–MNase), derived from both H1-hESC and HFFc6 cell lines.

We used BEDTools merge^35^ with the parameters ‘–c 1 -o count’ to remove redundant anchors (based on their genomic position) and to find the number of merged anchors at each genomic location. The number of merged anchors in a given genomic locus reflected loop valency at this anchor. Using BEDTools multiinter (https://bedtools.readthedocs.io/en/latest/content/overview.html) we identified the anchors that were shared in one, two or three protocols (Fig. 5a–c and Extended Data Fig. 6a–e).

#### CUT&RUN, CUT&Tag and ChIP-Seq analysis

CUT&RUN data (HFFc6 H3K4me3, HFFc6 H3K27ac, H1-hESC CTCF, H1-hESC H3K4me3, H1-hESC H3K27ac) were generated in the laboratory of S. Henikoff and can be found on the 4DN Data Portal (https://data.4dnucleome.org/). CUT&Tag data (HFFc6 CTCF, HFFc6 SMC1) were generated in the laboratory of R. Maehr at the University of Massachusetts Medical School. Finally, ChIP-Seq data were downloaded from ENCODE. We processed raw fastq files for CUT&RUN and CUT&Tag data and downloaded already processed bigwig and peak lists for ChIP-Seq data. We mapped and processed the fastq files using nf-core ATAC-Seq^36^ pipelines. BWA was used for mapping the fastq files to the hg38 reference genome; MACS2 (with default parameters) was used to find the enriched peaks, and BEDTools intersect was subsequently used to identify the loop anchors from these enriched peaks.

We found the anchors that intersected the three protocols (FA–DpnII, FA + DSG–DpnII, FA + DSG–MNase) and the FA + DSG–MNase-specific anchors using BEDTools intersect. We extracted the open chromatin (ATAC-Seq peak) regions located at these anchors and then aggregated the average signal enrichments of CTCF, SMC1, H3K4me3, H3K27ac, YY1 and RNA PolII. Deeptools was used to create the enrichment profiles in Fig. 5e and Extended Data Fig. 6f (ref. ^37^). We downloaded the lists of cCREs for H1-hESCs and HFFc6 cells from ENCODE^25^ and overlapped these cCREs with the intersected anchor list and the FA + DSG–MNase anchor list, again using BEDTools intersect. Finally we separated them based on the cCRE categories.

To compare the anchor-specific enrichments shown in Fig. 5g and Extended Data Fig. 6h, we used the loop lists of FA–DpnII, FA + DSG–DpnII and FA + DSG–MNase. We identified enriched convergent CTCF sites located at these loop anchors and compared the enrichments of CTCF, SMC1, H3K4me3, H3K27ac, YY1 and RNA PolII per anchor. To obtain convergent CTCF sites, we selected anchor 1 (left anchor) to overlap with CTCF sites that had a ‘+’ orientation and a CTCF peak, and anchor 2 (right anchor) to overlap with CTCF sites that had a ‘–’ orientation. We plotted convergent CTCF sites located at anchor 1 and anchor 2 for FA–DpnII, FA + DSG–DpnII and FA + DSG–MNase in both HFFc6 cells and H1-hESCs (Fig. 5f and Extended Data Fig. 6h).

For HFFc6 cells, we used CUT&Tag data generated with an antibody against the N terminus of CTCF. For H1-hESCs, we used CUT&RUN data generated with an antibody against the C terminus of CTCF. Given that CTCF motifs are known to locate at the N terminus of the CTCF protein^26^, the orientation of the CTCF enrichments differed between the datasets from CUT&Tag and CUT&RUN.

#### Insulation score

We calculated diamond insulation scores using cooltools (https://github.com/open2c/cooltools/blob/master/cooltools/cli/diamond_insulation.py) as implemented from Crane et al.^30^. We defined the insulation and boundary strengths of each 10-kb bin by detecting the local minima of 10-kb binned data with a 200-kb window size. We used the cooltools function diamond-insulation with the parameters –ignore-diags 2, –window-pixels 20. We separated weak and strong boundaries using the mean insulation score of each protocol (that is, weak boundaries < mean < strong boundaries). Given that diamond insulation pipelines cannot differentiate between compartment boundaries and insulation boundaries, we manually removed the compartment boundaries before any further analysis. Therefore the depth in local minima here is a result of strong insulation strength not a compartment switch. Next, we aggregated the insulation strength of the deep datasets at loop anchors, strong boundaries, and loop anchors located at the strong boundaries using scripts from the hic-data-analysis-bootcamp notebook (https://github.com/hms-dbmi/hic-data-analysis-bootcamp/blob/master/notebooks/06_analysis_cooltools-snipping-pileups.ipynb). For both deep and matrix data we used only strong boundaries for further analysis because they reflected the true boundaries across protocols. Given that the position of insulation boundaries was often offset by one or two bins between protocols, we extended the boundary bin by 10 kb on each side (30 kb total) in each protocol. We then used BEDTools multiinter (https://bedtools.readthedocs.io/en/latest/content/overview.html) to count the boundaries that were found in one or more protocols within the cell type. We defined our stringent boundary list as the boundaries that were shared in at least 50% of the matrix protocols within each cell type and used these boundary lists for further comparisons. In heatmaps, we used the average insulation strength of these boundaries per protocol (Extended Data Fig. 7k). To create the heatmaps in Extended Data Fig. 7l, we used the loop anchors that were shared between the three protocols that were deeply sequenced: FA + DpnII, FA + DSG–DpnII and FA + DSG–MNase in both H1-hESCs and HFFc6 cells.

#### Loop quantification for specific genomic separations

To quantify the loop strengths for HFFc6 deep datasets described in Fig. 6d (FA–DpnII, FA + DSG–DpnII, FA + DSG–DdeI, FA + DSG–DdeI + DpnII, FA + DSG–MNase), first we separated the loops based on their genomic separations into 100-kb bins, starting from 70 kb (that is, 70–170 kb, 170–270 kb,…970–1,070 kb), because 70 kb was the smallest detectable loop size, and then plotted the number of loops detected in each distance interval (Fig. 6d). Given that the number of detected loops in these genomic separations was different for each library, we sampled 1,000 loops for each distance from the FA + DSG–DdeI + DpnII dataset to quantify the loop enrichments of the five libraries (Fig. 6e). If the number of loops at a specified distance is smaller than 1,000 we use the entire loop set at this distance.

Finally, to create Fig. 6h,i we sampled 2,000 loops from each HFFc6 deep dataset (FA–DpnII, FA + DSG–DpnII, FA + DSG–DdeI, FA + DSG–DdeI + DpnII, FA + DSG–MNase), combined them and then quantified the loop strength of the total 10,000 loops in these deep datasets (Fig. 6h) and in the matrix datasets described in Fig. 1a (Fig. 6i). Loop enrichments were quantified as described in the Quantification of Chromatin Loops section.

#### Sampling experiment

We combined two biological replicates for the deep datasets obtained with each of the protocols. We then sampled 10 experiments with different numbers of interactions (valid pairs): 200 million reads, 400 million,…1,800 million, 2 billion reads. For each sample we then called and quantified compartment strength and loops, exactly as described above.

### Reporting Summary

Further information on research design is available in the Nature Research Reporting Summary linked to this article.

## Online content

Any methods, additional references, Nature Research reporting summaries, source data, extended data, supplementary information, acknowledgements, peer review information; details of author contributions and competing interests; and statements of data and code availability are available at 10.1038/s41592-021-01248-7.

## Supplementary information

Supplementary Fig. 1 Supplementary Fig. 1

Reporting Summary

Supplementary Table 1 Mapping statistics of all experiments used in this study

## Data Availability

Data are available at GEO under accession number GSE163666. Supplementary Table 1 lists datasets accessible through the 4DN data portal including 4DN accession numbers. Source data are provided with this paper.

## Source data

Source Data Fig. 2 Data points of cis/trans ratio, scaling plots and *P*(s) average slope for HFF.
Source Data Fig. 3 Compartment strength of all protocols.
Source Data Fig. 4 Loop strength of intersection and union loop lists.
Source Data Fig. 5 Loop and anchor relationship, protocol-specific and shared loop anchor lists.
Source Data Fig. 6 Data points of compartment strength and loop strength including Hi-C 3.0.
Source Data Extended Data Fig. 1 Fragment size distributions for cumulative plot and HiCRep correlations.
Source Data Extended Data Fig. 2 Data points of cis/trans ratio, scaling plots, *P*(s) average slope and mitochondrial interactions for all cell types.
Source Data Extended Data Fig. 3 Compartment strength of all protocols, first eigenvector correlations and coverages data points.
Source Data Extended Data Fig. 5 Loop strength of intersection and union loop lists.
Source Data Extended Data Fig. 6 Loop and anchor relationship, protocol-specific and shared loop anchor lists.
Source Data Extended Data Fig. 7 Number of commonly detected insulation boundaries and the strength of these boundaries.
Source Data Extended Data Fig. 8 Fragment size distributions for cumulative plot including Hi-C 3.0.
Source Data Extended Data Fig. 9 Spearman correlation of first eigenvectors, compartment strengths and number of detected loops for sampled experiments.

## Extended data

are available for this paper at 10.1038/s41592-021-01248-7.

## Code availability

Scripts and notebooks used in this paper can be accessed at https://github.com/dekkerlab/matrix_paper
