# Supplementary material for: Systematic evaluation of chromosome conformation capture assays
Source: Nat Methods. 2021 Sep 3;18(9):1046–55. doi: 10.1038/s41592-021-01248-7 (PMC8446342; doi:10.1038/s41592-021-01248-7)

nature portfolio

https://doi.org/10.1038/s41592-021-01248-7

Supplementary information

Systematic evaluation of chromosome conformation capture assays

In the format provided by the
authors and unedited

Supplementary Figure 1

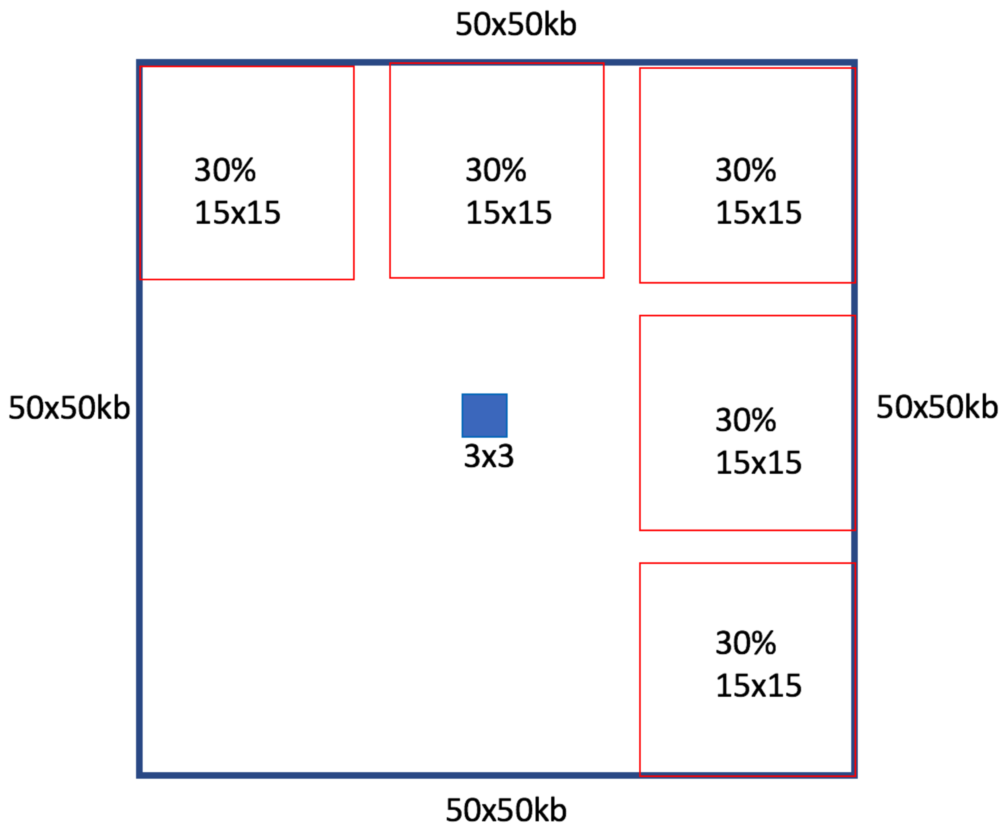

Supplement: Supplementary file 1 — Supplementary Fig. 1 [file 41592_2021_1248_MOESM1_ESM.pdf]
