# Supplementary material for: Systematic evaluation of chromosome conformation capture assays
Source: Nat Methods. 2021 Sep 3;18(9):1046–55. doi: 10.1038/s41592-021-01248-7 (PMC8446342; doi:10.1038/s41592-021-01248-7)
Supplement: Supplementary file 2 — Reporting Summary [file 41592_2021_1248_MOESM2_ESM.pdf]

nature research

Corresponding author(s): Job Dekker, Johan Gibcus

Last updated by author(s): Jul 8, 2021

Reporting Summary

Nature Research wishes to improve the reproducibility of the work that we publish. This form provides structure for consistency and transparency in reporting. For further information on Nature Research policies, see our Editorial Policies and the Editorial Policy Checklist.

Statistics

For all statistical analyses, confirm that the following items are present in the figure legend, table legend, main text, or Methods section.

n/a	Confirmed	
☒	☐	The exact sample size (*n*) for each experimental group/condition, given as a discrete number and unit of measurement
☐	☒	A statement on whether measurements were taken from distinct samples or whether the same sample was measured repeatedly
☒	☐	The statistical test(s) used AND whether they are one- or two-sided<br>*Only common tests should be described solely by name; describe more complex techniques in the Methods section.*
☒	☐	A description of all covariates tested
☒	☐	A description of any assumptions or corrections, such as tests of normality and adjustment for multiple comparisons
☒	☐	A full description of the statistical parameters including central tendency (e.g. means) or other basic estimates (e.g. regression coefficient) AND variation (e.g. standard deviation) or associated estimates of uncertainty (e.g. confidence intervals)
☒	☐	For null hypothesis testing, the test statistic (e.g. *F*, *t*, *r*) with confidence intervals, effect sizes, degrees of freedom and *P* value noted<br>*Give P values as exact values whenever suitable.*
☒	☐	For Bayesian analysis, information on the choice of priors and Markov chain Monte Carlo settings
☒	☐	For hierarchical and complex designs, identification of the appropriate level for tests and full reporting of outcomes
☒	☐	Estimates of effect sizes (e.g. Cohen's *d*, Pearson's *r*), indicating how they were calculated

Our web collection on statistics for biologists contains articles on many of the points above.

Software and code

Policy information about availability of computer code

Data collection	Distiller, Pairtools 0.3.0, Cooltools 0.2.0, Cooler 0.8.6, Higlass, Bedtools 2.28.0, PROSize3, java/1.8.0_77, singularity-3.2.0, nf-core, deeptools.
Data analysis	All code are available at a the following github repository that only contains the exact versions of each script used in this manuscript https://github.com/dekkerlab/matrix_paper

For manuscripts utilizing custom algorithms or software that are central to the research but not yet described in published literature, software must be made available to editors and reviewers. We strongly encourage code deposition in a community repository (e.g. GitHub). See the Nature Research guidelines for submitting code & software for further information.

Data

Policy information about availability of data

All manuscripts must include a data availability statement. This statement should provide the following information, where applicable:
- Accession codes, unique identifiers, or web links for publicly available datasets
- A list of figures that have associated raw data
- A description of any restrictions on data availability

Data are available at GEO under accession number GSE163666. Supplemental table 1 list datasets accessible through the 4DN data portal includes 4DN accession numbers.
https://www.ncbi.nlm.nih.gov/geo/query/acc.cgi?acc=GSE163666
https://data.4dnucleome.org/
https://www.encodeproject.org/files/ENCFF371JOS/@@download/ENCFF371JOS.bigWig
https://www.encodeproject.org/files/ENCFF609AFO/@@download/ENCFF609AFO.bigWig
https://www.encodeproject.org/files/ENCFF043CXM/@@download/ENCFF043CXM.bigWig

https://www.encodeproject.org/files/ENCFF515UYM/@@download/ENCFF515UYM.bigWig
https://www.encodeproject.org/files/ENCFF975VIV/@@download/ENCFF975VIV.bigWig
https://www.encodeproject.org/files/ENCFF580QUY/@@download/ENCFF580QUY.bigWig

Field-specific reporting

Please select the one below that is the best fit for your research. If you are not sure, read the appropriate sections before making your selection.

- [x] Life sciences
- [ ] Behavioural & social sciences
- [ ] Ecological, evolutionary & environmental sciences

For a reference copy of the document with all sections, see nature.com/documents/nr-reporting-summary-flat.pdf

Life sciences study design

All studies must disclose on these points even when the disclosure is negative.

Sample size	For each 3C-based assay at least 5 million cells were sampled.
Data exclusions	No data were excluded.
Replication	For experiment where 3C protocol parameters were tested, we obtained 1 replicate for each cell line (HFF, H1-hESC, H1 Derived Endoderm, HeLa S3 non-synchronized, HeLa S3 G1 and HeLa S3 arrested in prometaphase), so that a total of 6 experiments were obtained from 6 cell lines using the same 3C protocol parameters. Results were found to be reproducible across the set of 6 replicates.<br>For ultra-deeply sequenced data sets we obtained 2 biological replicates for each of the cell lines (HFFc6, H1-ESC).
Randomization	Randomization of this study was not necessary as we did not allocate datasets into experimental groups.
Blinding	Blinding of results were no necessary as the results are directly linked to the data.

Reporting for specific materials, systems and methods

We require information from authors about some types of materials, experimental systems and methods used in many studies. Here, indicate whether each material, system or method listed is relevant to your study. If you are not sure if a list item applies to your research, read the appropriate section before selecting a response.

Materials & experimental systems

n/a	Involved in the study	
☐	☒	Antibodies
☐	☒	Eukaryotic cell lines
☒	☐	Palaeontology and archaeology
☒	☐	Animals and other organisms
☒	☐	Human research participants
☒	☐	Clinical data
☒	☐	Dual use research of concern

Methods

n/a	Involved in the study	
☐	☒	ChIP-seq
☒	☐	Flow cytometry
☒	☐	MRI-based neuroimaging

Antibodies

Antibodies used	SMC1 (Bethyl, cat# A300-055A)<br>CTCF antibody (Active motif, cat # 61311)<br>secondary antibody (guinea pig α-rabbit antibody, cat. # ABIN101961), H3K4me3 Active Motif 39159, H3K27ac Millipore MABE647, CTCF Millipore 07-729.
Validation	All primary antibodies were shown to react to the known proteins on the manufacturer websites.

Eukaryotic cell lines

Policy information about cell lines

Cell line source(s)	Human Foreskin Fibroblasts, hTert immortalized (gift from Galloway lab, FHCRC, Seattle)<br>Human Foreskin Fibroblast, hTert immortalized clone 6 (clonal derivative of Human Foreskin Fibroblasts listed above); Dekker lab (Umass Medical School)<br>H1-ESC: WiCell<br>Endoderm cells derived from H1-ESC listed above: generated by Maehr lab (Umass Medical School)<br>HeLa S3 cells: ATCC CCL-2.2

Authentication	HFF and HFFc6: cell morphology<br>H1-ESC: cell morphology and pluripotency marker expression.<br>Endoderm: endoderm marker expression<br>HeLa S3: Hi-C based karyotyping
Mycoplasma contamination	All cells were regularly tested and confirmed to be free of mycoplasma
Commonly misidentified lines<br>(See ICLAC register)	No commonly misidentified cell lines were used.

ChIP-seq

Data deposition

☒ Confirm that both raw and final processed data have been deposited in a public database such as GEO.

☒ Confirm that you have deposited or provided access to graph files (e.g. BED files) for the called peaks.

Data access links<br>*May remain private before publication.*	Data are available at GEO under accession number GSE163666. Supplemental table 1 lists datasets accessible through the 4DN data portal includes 4DN accession numbers.
Files in database submission	Supplemental table 1
Genome browser session<br>(e.g. UCSC)	HFFc6: http://matrix-review.dekkerlab.org:8080/l/?d=MFWVbiCGSAyv5FInIRF7_Q<br>H1-hESC: http://matrix-review.dekkerlab.org:8080/l/?d=XJCWFbpNRHe2VVHJUThYcQ

Methodology

Replicates	2 replicates of each CTCF, SMC1 Cut&Tag Datasets for HFFc6, 2 replicates of ATAC-Seq for HFFc6, 4 replicates of Cut&Run datasets for HFFc6 targeting H3K4me3, 4 replicates of Cut&Run datasets for HFFc6 targeting H3K27Ac.
Sequencing depth	Stats for combined biological replicates:<br>Experiment : Total_number_of_reads - uniquely_mapped_reads - read_length - single or paired_end<br>HFFc6 Atac-seq : 360,005,92 - 353,672,578 - 50 - paired_end<br>HFFc6 SMC1 : 61,975,832 - 61,940,464 - 40 - paired_end<br>HFFc6 CTCF : 70,833,504 - 70,787,166 - 40 - paired_end<br>HFFc6 H3K4me3 : 72,872,038 - 64,865,180 - 25 - paired_end<br>HFFc6 H3K27Ac : 65,304,704 - 52,091,930 - 25 - paired_end
Antibodies	Cut&Tag experiments CTCF antibody from Active motif, cat # 61311, SMC1 antibody from Bethyl, cat# A300-055A.<br>Cut&Run experiments H3K4me3 antibody from Active Motif cat # 39159, H3K27Ac antibody from Millipore cat # MABE647.
Peak calling parameters	bwa=0.7.17 is used for mapping and macs2=2.2.7.1 is used for peak calling with default parameters.<br>Details about the parameters can be found here: https://github.com/nf-core/atacseq/blob/master/nextflow.config
Data quality	FastQC (https://www.bioinformatics.babraham.ac.uk/projects/fastqc/) is performed to check the quality of the sequencing files (Base quality, redundancy,..etc). The number of peaks (FDR 5%) called in HFFc6 Atac-seq is 156,280 and in HFFc6 CTCF is 97,688.
Software	https://nf-co.re/atacseq/1.2.1 is used to map and process the data.
